# Germplasm Mining of *Prunus domestica* L.: Multi-Year Assessment of Pomological Characters to Identify Candidate Elite Donor Parents for European Plum Breeding and Their Genetic Evaluation

**DOI:** 10.3390/plants15132095

**Published:** 2026-07-06

**Authors:** Michaela Marklová, Liliia Pavliuk, Jana Čmejlová, Boris Krška, Jiří Sedlák

**Affiliations:** Research and Breeding Institute of Pomology Holovousy Ltd., Holovousy 129, 508 01 Hořice, Czech Republic; michaela.marklova@vsuo.cz (M.M.); liliia.pavliuk@vsuo.cz (L.P.); jana.cmejlova@vsuo.cz (J.Č.); boris.krska@vsuo.cz (B.K.)

**Keywords:** *Prunus domestica*, European plum, germplasm, donor parents, pre-breeding, pomological traits, fruit quality, trait correlations, stability across years, SSR marker, parentage analysis, PCA, DAPC, Bruvo’s distance, dendrogram, tanglegram

## Abstract

European plum (*Prunus domestica* L.) breeding for competitive production increasingly requires donor parents that combine attractive, market-oriented fruit quality with stable trait expression. This study evaluated a set of 36 phenotypically highly different cultivars from the germplasm collection maintained at the Research and Breeding Institute of Pomology Holovousy Ltd. (the Czech Republic). First, genetic analyses based on SSR marker data were performed to assess the diversity and kinship relationships within the selected collection of plum varieties. Several parentage combinations were successfully identified for cultivars with previously undocumented origins. Population-level analyses confirmed broad genetic diversity and separated the collection into four genetically distinct groups. Phenotypes were obtained on fruits ripened on trees from a non-irrigated orchard on myrobalan rootstock over five consecutive years (2019–2023). Pomological and related quality traits were recorded using nine-point UPOV-based rating scales together with instrumental measurements. The dataset included fruit size and shape descriptors, skin and flesh color, wax bloom, soluble solids (°Brix), firmness, bruising resistance, stone separability, and sensory attributes (flavor, aroma, juiciness, texture, and acidity). Interannual variability was quantified using coefficients of variation, and relationships among traits were explored using Pearson correlations. The results revealed broad phenotypic diversity among the individual varieties and also their genetic groups. Finally, phenotypes were associated with genotypes, and the most genetically determined traits were identified. Multi-year stability profiling supported the identification of candidate elite donor cultivars that combine favorable attributes for the fresh market and/or traits relevant to processing. These findings provide a practical pre-breeding shortlist and quantitative trait targets to support crossing design and selection under central European conditions.

## 1. Introduction

European plum (*Prunus domestica* L.) is a long-lived, deciduous fruit tree species of the genus *Prunus* (*Rosaceae*), cultivated in temperate regions for its fleshy drupe, which is used for both fresh consumption and processing [1,2,3]. The species is predominantly propagated vegetatively in commercial orchards and extensive breeding programs are underway worldwide with the intention to improve the existing varieties. However, long breeding cycles present a significant challenge for breeders. Therefore, robust multi-year phenotyping is essential for deciding which variety to plant in an orchard or use as a parent in crossings [4]. From a cytogenetic perspective, *P. domestica* is a hexaploid species (2n = 6× = 48), a feature that has major implications for trait inheritance, segregation patterns, and the design of both phenotypic and molecular characterization pipelines [3,5], including complex allele dosage effects, non-Mendelian segregation patterns, and increased requirements for robust molecular marker systems. Recent studies have provided evidence that supports the long-standing hypothesis that the European plum originated through interspecific hybridization involving the diploid species *Prunus cerasifera* Ehrh. and tetraploid species *Prunus spinosa* L., followed by stabilization and diversification under cultivation and human-mediated dispersal [3,6].

In horticultural practice, ‘plums’ represent a diverse pomological complex with substantial variation in fruit size, shape, skin and flesh color, stone adherence, ripening window, and suitability for targeted end uses [1,7]. This diversity is reflected in the traditional grouping of domestica-type plums into multiple market- and processing-relevant forms, which remain important for cultivar typology and breeding priorities in Europe [1,8,9]. The term plums (*P. domestica*) refers to the pomological group that includes plums, semi-plums, damsons, gages, and mirabelles. In central and eastern Europe, the cultivation of European plum has a long history and remains highly relevant from a socioeconomic standpoint due to its role in both the supply of fresh fruit and processing chains. However, production systems are increasingly exposed to climatic volatility and shifting consumer expectations for consistent quality [10,11].

European plums exhibit extensive diversity, both phenotypically and genetically. Determining the true identity of varieties is essential for cultivating and breeding plum trees. Due to their hexaploid nature, plum accessions are most commonly analyzed using SSR markers [12], as SNP genotyping requires high sequencing depth to avoid allele dropout, and advanced biostatistical approaches to interpret sequencing data [13]. Several plum collections have been characterized using different SSR marker sets. For example, Urresterazu et al. [14] investigated the genetic diversity of Spanish and other European plum accessions. Makovics Zsohár et al. [15] evaluated Hungarian germplasm, and Manco et al. [16] analyzed Italian accessions. In 2020, the European Cooperative Programme for Plant Genetic Resources recommended a set of nine SSR markers for European plum genotyping [17], each of which was amplified in a separate PCR reaction. This recommended SSR set was subsequently applied, for example, by Meland et al. [18] to genetically characterize Norwegian plum germplasm. More recently, Čmejlová et al. [5] simplified European plum fingerprinting by multiplexing eight highly polymorphic and easily interpretable SSR markers into a single PCR and fragment analysis reaction. This eight-plex can be supplemented by the multiplex of four other SSR markers to increase the robustness of genetic studies. SSR data are most frequently used for cultivar identification and for assessing population diversity and constructing dendrograms [5,14,15,18], whereas parentage analysis is performed only rarely [18,19].

The main aim of our work was to comprehensively characterize candidate parents suitable for European plum (*Prunus domestica*) breeding, with the goal of selecting elite varieties that could serve as the foundation of a core breeding collection. Thirty-six varieties representing broad phenotypic diversity within the RBIP Holovousy germplasm collection were selected, and clonal varieties were included as controls. First, the genetic diversity of the selected material was assessed. Using SSR markers, parentage analysis was conducted to examine close relationships among the varieties. Next, population-level analyses were performed to further confirm or refute substantial genetic distances or relatedness among individual accessions and to divide the collection into interpretable groups. A multi-year field evaluation was then carried out to characterize pomological traits in detail. Trait stability across years and their mutual correlations were statistically evaluated, as were differences among the groups previously defined by genetic analyses. Cluster analysis using phenotypic data was done and compared with the results obtained with molecular markers. Finally, candidate elite donor parents were identified for breeding programs aimed at developing new table and/or processing cultivars.

## 2. Results

### 2.1. Genetic Analyses

#### 2.1.1. Parentage Analysis

The parental combinations for the selected collection of 36 varieties were searched for in the database of 242 unique genotypes described by Čmejlová et al. [5], and were then compared with the published parental origins of the individual varieties [20] (Table 1—an overview; Supplementary Table S1—detailed analyses). In cases where only one parent was known/analyzed, the samples were compared manually. Since there are many varieties with clonal variants that differ by 1–2 alleles [5], parental combinations that differ in the offspring-parent_1-parent_2 trio by one allele were also accepted.

For most varieties, the proposed parental combinations corresponded to those published. One exception was ‘Cacanska Lepotica’ (c), for which the published origin was not confirmed. Instead, POLYGENE identified ‘Ruth Gerstetter’ and ‘Stanley’ (c) as possible parents of the variety rather than the ‘Wangenheim’ × ‘Pozegaca’ cross. For several varieties, a different second parent was identified than was previously known. For example, ‘Carpatin’ was found to have ‘Ruth Gerstetter’ as the second parent. However, the original ‘Early Rivers’ variety was not tested. ‘Auerbacher’ was identified as the parent of ‘Topking’ instead of ‘Italian Prune’, which was analyzed. Additionally, ‘Cacanska Najbolja’ may be the parent of ‘Toptaste’ instead of ‘German Prune’, which was not tested. Additionally, ‘Topstar’ is a potential sibling of ‘President’ rather than ‘Ersinger’. For ‘Gabrovska’, ‘Svestka domaci’ was proposed instead of ‘Kjustendilska modra’, which was not analyzed. However, it should be a clone of ‘Svestka domaci’. A pollinator for the ‘Althanova Renkloda’ was proposed to generate ‘Excalibur’; it could be ‘Washington’. For ‘Top 2000’, it can be assumed that the second parent, which was previously unknown, is ‘Auerbacher’. Parent combinations were also proposed for varieties of previously unknown origin. ‘Anna Spaeth’ may have originated from ‘Zelena Renkloda’ × ‘Svestka domaci’. ‘Dambovita’ may be an offspring of a cross between ‘Tuleu Gras’ and ‘Malvazinka’, and ‘Viola Szini Diapre’ and ‘Svestka domaci’ may be the parents of ‘Esslingenska svestka’. Additionally, ‘Belgicka modra’, ‘Bystricka muskatova’, and ‘Svestka domaci’ were identified as clones differing by one or two alleles in the 12 SSR markers analyzed [5], as were ‘Mirabelka Metska’ and ‘Mirabelka Nancy’.

Taken together, two parents were suggested for 16 cultivars, and one parent was confirmed in further three varieties. Twenty out of 35 suggested parents represented different accessions; ‘Cacanska Najbolja’ (5×) and ‘Stanley’ (c) (5×) were the most frequently crossed parent accessions in this collection.

#### 2.1.2. Analysis of Genetic Diversity

**Figure 1:**
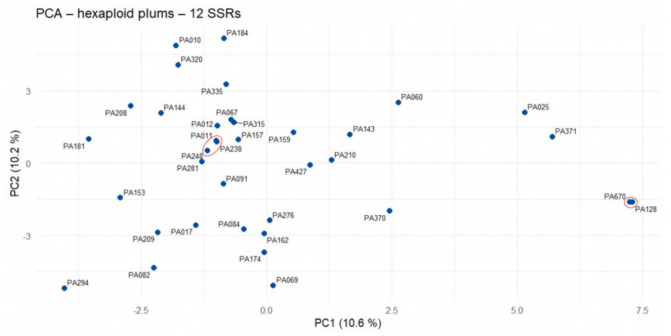
PCA of the presented hexaploid plum collection. The codes of individuals correspond to Table 1; the observed clones are circled by red ellipses.

The genetic structure of the collection was analyzed using Principal Component Analysis (PCA) based on data coming from the allelic compositions of 12 SSR markers [5]. PC1 and PC2 explained only 10.6% and 10.2% of the total variance, respectively (Figure 1). The accessions were disseminated throughout the plot area. The only exceptions were the samples PA670 (‘Mirabelka Nancy’) and PA128 (‘Mirabelka Metska’); and PA011 (‘Bystricka muskatova’), PA238 (‘Svestka domaci’), and PA248 (‘Belgicka modra’), which closely colocalized in the PCA, confirming their clonality (red ellipses in Figure 1). The close colocalization of PA067 (‘Cacanska Lepotica’ (c)) and PA315 (‘Top 2000’) may be explained by their similar origin: ‘Stanley’ (c) is a parent of both these varieties.

**Figure 2:**
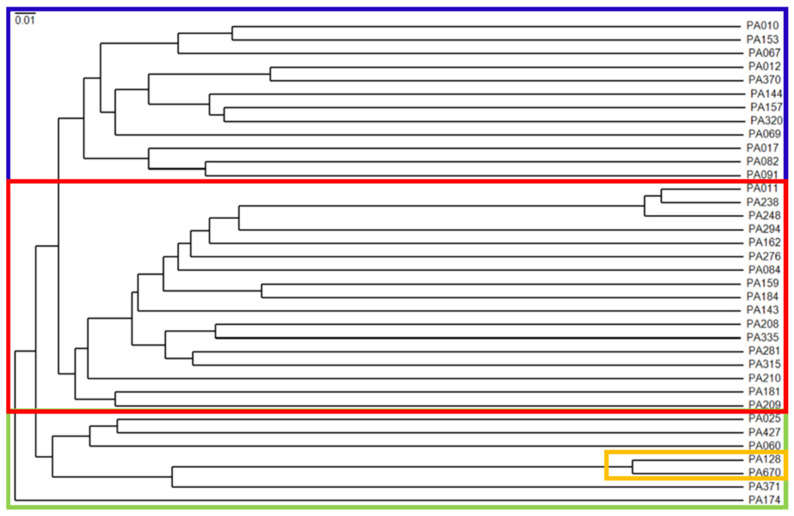
The dendrogram for the analyzed collection with manually imputed rectangles dividing the tree into four groups. The orange rectangle was added based on the DAPC because PA128 (‘Mirabelka Metska’) and PA670 (‘Mirabelka Nancy’) clearly separated at all K values tested in the DAPC (K = 3–5, Supplementary Table S2). The codes of the individuals correspond to Table 1.

**Figure 3:**
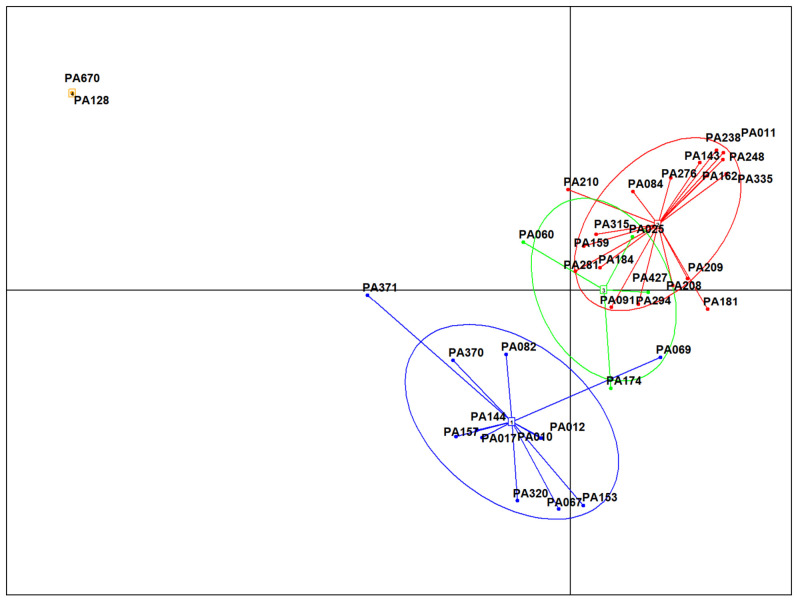
DAPC with the number of clusters K = 4 showing the Orange group of mirabelles, ‘Metska’, and ‘Nancy’; Green group of gages and mirabelles; Blue group of modern cultivars; and Red group of landraces and Balkan and Romanian modern cultivars. The codes of the individuals correspond to Table 1.

Since PCA only explained a small proportion of the total variance, Discriminant Analysis of Principal Components (DAPC) was also applied. This analysis maximizes between-group variation and provides clearer clustering patterns than PCA. However, the automated selection of the number of clusters using the Bayesian Information Criterion preferred K = 1, indicating weak genetic structure in the dataset as expected for genetically diverse core collections. Therefore, a combination of results obtained from hierarchical clustering (dendrogram) and DAPC with different K values tested was used to identify biologically interpretable groups. The dendrogram (Figure 2) revealed a natural division into three branches (blue, red, and green rectangles). The fourth orange rectangle was added based on the DAPC because PA128 (‘Mirabelka Metska’) and PA670 (‘Mirabelka Nancy’) clearly separated at all K values tested in the DAPC (K = 3–5, Supplementary Table S2). Based on cluster stability, size, and biological interpretability, K = 4 was finally selected as the most appropriate for the DAPC (Figure 3). The results for K = 3 and K = 5 can be found in Supplementary Table S2. At this K = 4, the dendrogram and the DAPC produced very similar distribution of all accessions into groups with only a few exceptions.

The Blue group of the dendrogram consists predominantly of modern European and North American varieties, many of which share ancestry through widely used founders such as ‘Stanley’ (c) and related large-fruited cultivars. Examples include ‘Stanley’ (c), ‘Valor’, ‘Herman’, ‘Topstar’, ‘Topgigant’, ‘Cacanska Lepotica’ (c), ‘Timocanka’, ‘Toptaste’, and ‘Aprimira’. In contrast, the Red group consists of traditional landraces such as ‘Svestka domaci’ and its clones, ‘Anna Spaeth’, ‘Ortenauer’, ‘Esslingenska svestka’, ‘Chodovlicka’, ‘Vankova Chrudimska’, and several Balkan and Romanian cultivars including ‘Gabrovska’, ‘Valjevka’, and ‘Tuleu Gras’. The Green group contains gages and mirabelles, as well as ‘Opal’, which is the result of crossing ‘Oullins Renclode’ and ‘Bellamira’ with ‘Mirabelka Nancy’ as one parent. The Orange subgroup contains two mirabelles, ‘Metska’ and ‘Nancy’.

The DAPC revealed a nearly identical division into the groups (the clusters in Figure 3 are colored the same as the groups in the dendrogram). There were only two exceptions. The first one was PA371 (‘Bellamira’), likely due to its parentage: the modern, large-fruited ‘Cacanska Najbolja’ × ‘Mirabelka Nancy’. The DAPC assigned ‘Bellamira’ to the Blue group of modern cultivars instead of the Green group of gage/mirabelle cultivars in the dendrogram. The second exception was PA091 (‘Carpatin’), which was found in the Red DAPC group of landraces and accessions originating from the Balkans and Romania instead of the Blue dendrogram group containing modern genotypes. However, ‘Carpatin’ is a modern large-fruited accession from Romania so its fluctuation between the two groups is justifiable.

**Figure 4:**
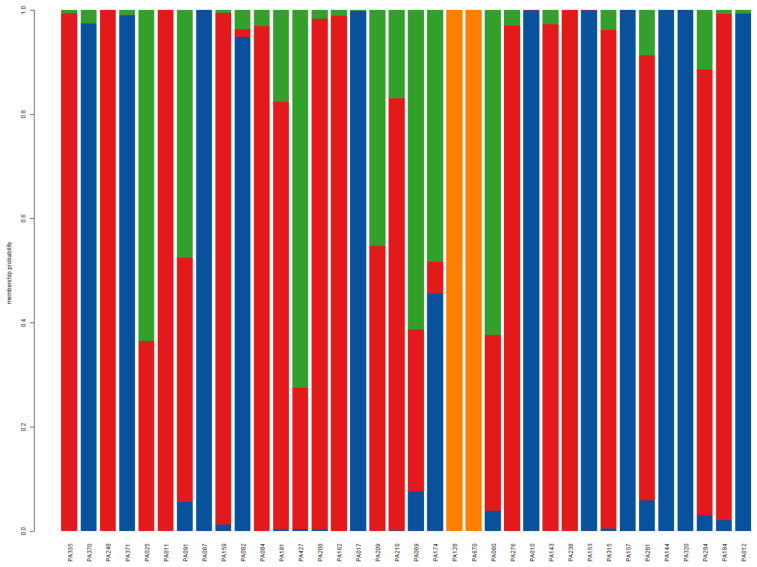
The compoplot showing the proportional membership of each accession in the genetic clusters in Figure 3 (the same colors were used). The codes of the individuals correspond to Table 1.

Along with the DAPC, a compoplot was generated to display the proportional membership of each accession in the genetic clusters (Figure 4). The plot clearly shows the genetic separation of PA128 (‘Mirabelka Metska’) and PA670 (‘Mirabelka Nancy’), with no admixture from other clusters. Notably, only minimal admixture is observed between the Blue and Red clusters, indicating a strong separation between these two genetic groups. In contrast, the Green cluster is only partially dominant within individual genotypes, reflecting the separation of these varieties from the Red and Blue genetic clusters detected in the DAPC with K = 3 (Supplementary Table S2).

**Figure 5:**
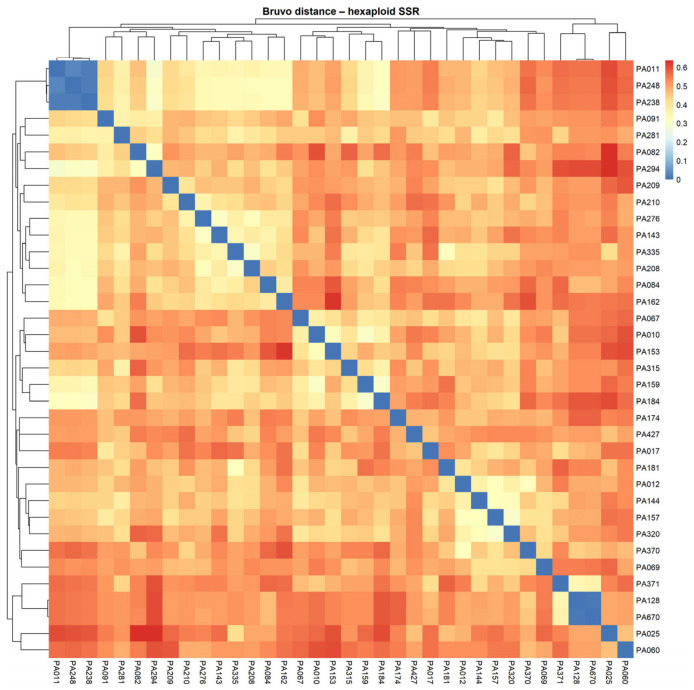
Heatmap based on Bruvo’s distance values. Blue squares out of the diagonal represent clonal varieties, and the reddest squares visualize the highest genetic distance between the two compared varieties. The codes of the individuals correspond to Table 1.

We also evaluated genetic diversity among individual accessions using Bruvo’s distance analysis and visualized the results as a heatmap (Supplementary Table S3, Figure 5). Bruvo’s distance ranges from 0 to 1, with 0 indicating identical genotypes and 1 representing a theoretical maximum. No universally accepted threshold values have been established, as Bruvo’s distance is strongly influenced by marker variability, ploidy level, and the genetic structure of the dataset [22,23].

**Figure 6:**
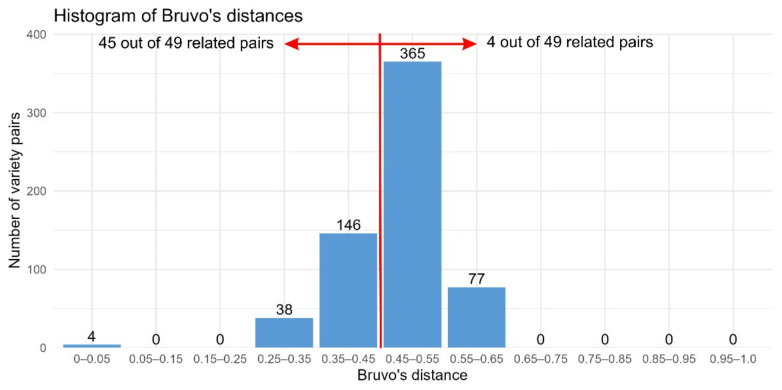
Histogram based on Bruvo’s distance values divided into 11 categories designed to best distinguish related variety pairs from unrelated ones (red line).

The analyzed variety collection was therefore evaluated *per se* using the histogram of Bruvo’s distances, which were divided into eleven meaningful categories (Figure 6). The results confirmed the clonality of ‘Belgicka modra’, ‘Bystricka muskatova’, and ‘Svestka domaci’, as well as of ‘Mirabelka Metska’ and ‘Mirabelka Nancy’, with Bruvo’s distances of 0.01–0.03. The threshold for clonal varieties was therefore set at 0.05. The following categories of the histogram were extended to 0.1 to reduce the number of categories. Non-clonal accessions exhibited Bruvo’s distances ranging from 0.297 (‘Toptaste’ vs. ‘Topgigant’, which share the same suggested parent, ‘Cacanska Najbolja’) to 0.637 for ‘Dambovita’ vs. ‘Bonne de Bry’. A detailed analysis of this data identified probable close relatives (parent–offspring pairs or siblings sharing at least one parent) as pairs with Bruvo’s distance values typically ranging from 0.25 to 0.45. A total of 184 variety combinations belong to this category; 45 out of 49 close relatives according to Table 1 fall into it. According to this criterion, the varieties with an unknown pedigree ‘Hamanova svestka’, ‘Ortenauer’ and ‘Schueleho rana’ seem to be close relatives of ‘Svestka domaci’ due to Bruvo’s distances bellow 0.4. The remaining 442 variety combinations showed Bruvo’s distances higher than 0.45, with a maximum observed value of 0.637. Only four out of 49 close relatives according to Table 1 fell into this category. More than 70% of variety pairs exhibited Bruvo’s distances above 0.45; the value established as the threshold of unrelatedness for the analyzed set of SSR markers. For example, the varieties ‘Mirabelka Jaune de Plovdiv’ and ‘Opal’ showed no Bruvo’s distance with other varieties bellow 0.45. The closest relative of ‘Bonne de Bry’ and ‘Mirabelka Flotova’ were ‘Anna Spaeth’ (0.426) and ‘Gabrovska’ (0.435), respectively.

To summarize the results of the genetic diversity analyses, all tests confirmed a broad genetic background of the chosen varieties. The result of automated selection of the number of clusters in DAPC K = 1 was especially important, as this suggests the non-existence of genetically distinguishable groups in the analyzed collection. Forced division of the collection identified four clusters (K = 4); these were used for the grouping of the analyzed varieties during phenotyping to compare not only individual accessions but also genetically different groups. The implemented clonal controls, although they differ in phenotype, clearly demonstrate the genetic boundaries of such close relatedness.

### 2.2. Phenotype Analyses

#### 2.2.1. External Pomological Traits

First of all, external pomological traits such as fruit shape, skin and flesh color, wax blooming, and fruit bruising resistance, were evaluated (Table 2).

Fruit shape displayed pronounced inter-cultivar variability within the evaluated *P. domestica* collection. Mean scores spanned nearly the entire UPOV-derived scale, highlighting the diverse pomological background of the studied germplasm. The lowest values, corresponding to nearly spherical fruits, were predominantly recorded in the mirabelle groups, specifically in ‘Mirabelka Flotova’, ‘Bonne de Bry’ and ‘Mirabelka Nancy’ (all 1.0 ± 0.0). In contrast, elongated-to-ovoid fruit shapes were typical of several widely grown and breeding-relevant cultivars, including ‘Cacanska Lepotica’ (c), ‘Carpatin’, ‘Bellamira’ and ‘Topgigant’ (all 7.0 ± 0.0), with the highest mean value observed in ‘Valjevka’ (7.2 ± 1.0). Intermediate morphotypes were common among traditional European plum cultivars such as ‘Stanley’ (c), ‘Cacanska rodna’, ‘Hamanova svestka’ and ‘Svestka domaci’ (Domestic plum), reflecting the continuum of shapes characteristic of domestica-type plums.

The fruit color exhibited a very wide range of values within the evaluated collection, reflecting the pronounced pomological diversity of the studied material. Scores on the nine-point scale ranged from the lowest category (2.0), which is typical of yellow-colored fruits of mirabelle cultivars such as ‘Mirabelka Flotova’, ‘Aprimira’ and ‘Mirabelka Jaune de Plovdiv’, to the highest category (8.0), which characterize the blue-purple skin color of several domestica-type plums. This group included 12 cultivars, among others, the control cultivars ‘Stanley’ (c) and ‘Cacanska Lepotica’ (c), as well as the market cultivars ‘Toptaste’ and ‘Topgigant’. Most commercially important cultivars were concentrated in the upper part of the scale.

Flesh color showed a narrower range of values and overall lower variability compared with skin color. The lowest scores (3.0), corresponding to very light green-yellow flesh, were recorded in cultivars such as ‘Cacanska Lepotica’ (c), ‘Bonne de Bry’ and ‘Cacanska rodna’. In contrast, higher ratings (6.8–7.0), indicative of flesh colored more intensely yellow-orange, were observed in genotypes including ‘Opal, ‘Mirabelka Flotova’, and ‘Aprimira’.

The visual perception of bloom intensity (wax coating) varied significantly among cultivars, as did the degree to which this trait was expressed. The lowest values (2.4 or 2.5) were observed in yellow-fruited cultivars ‘Mirabelka Jaune de Plovdiv’, ‘Aprimira’, and ‘Katalonsky spendlik’. At the opposite end of the spectrum, cultivars with pronounced bloom reached a value of 8.0 points. These included the blue-fruited ‘Stanley’ (c) and ‘Belgicka modra’.

Fruit bruising resistance exhibited pronounced variability within the evaluated collection and ranged from 2.8 to 8.0, indicating substantial differences in the mechanical resistance of fruits among cultivars. The lowest values, which correspond to a high susceptibility of fruits to mechanical damage, were recorded in several mirabelle and small-fruited genotypes. These included ‘Mirabelka Flotova’ (2.8), and ‘Mirabelka Jaune de Plovdiv’ (4.4). These cultivars were characterized by softer skin and lower bruising resistance. In contrast, the highest bruising resistance values (7.0–8.0 points), which characterize fruits with high mechanical resistance, were observed in several domestica-type cultivars, including ‘Hamanova svestka’ (8.0), ‘Valjevka’ (7.4), and the control cultivar ‘Cacanska Lepotica’ (c) (7.0 points). These cultivars exhibited greater firmness of surface tissues, a prerequisite for improved fruit handling of fruits during harvest, grading, and distribution [24,25]. Most commercially important cultivars were concentrated in the middle to upper part of the scale (6.0–7.0 points).

#### 2.2.2. Internal Structural Pomological Characteristics

Next, internal structural pomological traits such as skin firmness and separability, flesh firmness and texture, and stone separability were determined (Table 3).

Skin firmness exhibited moderate to high variability within the evaluated collection, ranging from 4.3 to 7.3 on the evaluation scale. The lowest values, corresponding to softer skin, were recorded for cultivars such as ‘Aprimira’ (4.3) and ‘Herman’ (4.6). In contrast, the highest skin firmness values were observed in the cultivars ‘Bellamira’ (7.3), ‘Dambovita’ (7.3), ‘Belgicka modra’, ‘Gabrovska’, ‘Topgigant’, ‘Tuleu Gras’, and the control cultivar ‘Cacanska Lepotica’ (c) (all 7.0 points). These results indicate an increased mechanical resistance to the surface damage.

Skin separability covered a broader range (3.0–7.2) and showed pronounced differences among cultivars. The lowest values, characterizing difficult skin separation, were recorded for ‘Belgicka modra’ (3.0), ‘Cacanska rodna’ (3.3), and ‘Hamanova svestka’ (3.4). Conversely, high detachability scores (6.6–7.2) were observed in cultivars such as ‘Herman’ (7.2), ‘Opal’ (7.0 points), and ‘Timocanka’ (6.6) indicating differences in the adhesion strength between the skin and the flesh across the evaluated genotypes.

Flesh firmness exhibited moderate variability within the evaluated collection, ranging from approximately 4.0 to 7.4 points. This indicates differences in the mechanical properties of the mesocarp among cultivars. The lowest values, which characterize softer flesh, were recorded for the ‘Mirabelka Flotova’ cultivar (4.0). In contrast, the highest flesh firmness values were observed in ‘Gabrovska’ (7.4) and ‘Bellamira’ (7.3), reflecting greater structural integrity of the flesh in these genotypes. Most commercially important cultivars were concentrated in the middle to upper part of the scale (5.5–6.5 points).

Fruit texture in the evaluated collection ranged from 4.0 to 7.0 points. The lowest value was recorded for ‘Bellamira’ (4.0). On the other hand, the highest values and fine texture were recorded for ‘Anna Spaeth’, ‘Carpatin’, and ‘Hamanova svestka’ (7.0). In the evaluated collection, stone separability, a key technological trait in European plums, ranged from 5.0 (‘Mirabelka Flotova’) to 9.0, meaning that no flesh remains on the stone (‘Bellamira’, ‘Bystricka muskatova’, ‘Gabrovska’, ‘Hamanova svestka’, ‘Opal’, and ‘Topstar’).

#### 2.2.3. Internal Organoleptic Pomological Characteristics

Internal organoleptic pomological traits belong to the most important fruit characteristics for a consumer and they were evaluated as the following features: orthonasal aroma of the flesh, taste of the skin, juiciness, taste of the fruit, retronasal aroma, and perceived acidity/sweetness balance (Table 4).

The orthonasal aroma of the flesh exhibited a moderate range of values across the evaluated cultivars, with mean scores ranging from 4.0 to 6.2 points. The lowest aroma intensity, 4.0, was recorded in ‘Cacanska Lepotica’ (c) and ‘Toptaste’, followed by the relatively low value 4.3 in ‘Stanley’ (c), ‘Cacanska rodna’ and ‘Schueleho rana’. In contrast, the highest orthonasal aroma scores, 6.0 and higher, were recorded in the cultivars ‘Aprimira’ (6.0), ‘Belgicka modra’ (6.0) and ‘Chodovlicka’ (6.2), indicating a more pronounced perception of flesh aroma in these genotypes. Overall, most cultivars were concentrated in the middle part of the scale, reflecting the moderate expression of orthonasal aroma of the flesh within the evaluated plum collection.

The taste of the skin displayed a moderate range of values across the evaluated cultivars, with mean scores ranging from 3.4 to 6.6 points. The lowest score, 3.4, was recorded in ‘Topking’ and followed by a relatively low score of 4.0 in ‘Topgigant’. In contrast, the highest skin taste scores were observed in ‘Opal’ and ‘Timocanka’ (6.6). Most cultivars were centered in the middle part of the scale, indicating predominantly acidic/sour to moderately sweet skin taste.

The juiciness of the evaluated cultivars showed a moderate range of values, with mean scores ranging from approximately 4.0 to 7.3 points. The cultivars ‘Topstar’ and ‘Bystricka muskatova’ had the lowest juiciness values, at 4.0 and 4.4, respectively. On the other hand, the highest juiciness score was recorded in ‘Dambovita’ (7.3). Overall, most cultivars were concentrated in the middle to upper part of the scale, indicating predominantly juicy to very juicy flesh.

The taste of fruit exhibited an above-average value across the evaluated cultivars, with mean scores ranging from 5.0 to 7.8 points. The lowest taste scores were recorded in ‘Mirabelka Nancy’ (5.0), followed by ‘Schueleho rana’ (5.3) and ‘Mirabelka Metska’ (5.5). The highest fruit taste scores were recorded in ‘Hamanova svestka‘(7.8), ‘Bellamira’ (7.7) and ‘Toptaste’ (7.6) indicating a more pronounced and favorable overall flavor perception in these cultivars. High scores in this trait reflect predominantly good fruit taste in the evaluated group.

The retronasal aroma score was also above average, ranging from 4.8 to 7.2 points. The lowest retronasal aroma scores, 4.8, were recorded for ‘Schueleho rana’ and ‘Mirabelka Nancy’, and ‘Topgigant’ had a score of 5.0. The highest scores, 7.2, were recorded for ‘Esslingenska svestka’ and ‘Hamanova svestka’, indicating a more pronounced perception of aroma during mastication in these cultivars. Overall, most cultivars were assembled in the slightly above-average range, reflecting predominantly well-developed aromas.

The perceived acidity/sweetness balance exhibited an above-average score across the evaluated cultivars, with mean scores ranging from 5.3 (‘Schueleho rana’) to 7.8 points for ‘Anna Spaeth’, ‘Aprimira’, ‘Excalibur’, and ‘Vankova Chrudimska’. This indicates that the evaluated cultivars predominantly taste slightly to very sweet.

#### 2.2.4. Measurable Pomological Traits

Fruit weight and dimensions (height, width, and thickness), length of pedicel, and soluble solids content were determined as measurable pomological traits (Table 5).

The fruit weight showed very high variability in the evaluated cultivar collection and ranged from 9.3 g (‘Mirabelka Metska’) to 78.7 g (‘Topgigant’). Most of the cultivars presented a weight between 30 and 50 g.

The individual dimensions differed by as much as twofold across the evaluated varieties. Fruit height ranged from 25.5 mm (‘Mirabelka Flotova’) to 58.2 mm (‘Topgigant’), fruit width from 24.5 mm (‘Mirabelka Metska’) to 48.9 mm (‘Ortenauer’), and fruit thickness from 24.0 mm (‘Mirabelka Metska’) to 48.1 mm (‘Excalibur’). However, the typical fruit height ranged from 40 to 50 mm, the most common width was approximately 30–35 mm, and the plums had a thickness about 35 mm.

Pedicel length, an important trait for mechanically assisted or fully automated harvesting systems, also varied greatly, ranging from 10.6 mm (‘Herman’) to 26.6 mm (‘Excalibur’). The usual length of the pedicel was in the range 17 to 20 mm. In plums, soluble solids content (SSC) primarily reflects soluble sugars (glucose, fructose, sucrose), but also includes sorbitol, organic acids (mainly malic acid), amino acids, phenolic compounds, vitamins and mineral ions. Soluble solids content measured as °Brix ranged from 12.0 to 24.8 across the evaluated cultivars, with the minimum recorded in ‘Herman’ and the maximum in ‘Topking’. Across the evaluated plum varieties, soluble solids content typically ranged from 17 to 22 °Brix.

### 2.3. Variability of Traits Across Years

All characteristics were evaluated over five consecutive years, and their interannual stability was expressed as the coefficient of variation (CV). All cultivars were classified into three categories: varieties with low to negligible variability (CV < 10%), cultivars showing moderate variability (CV 10–30%), and accessions exhibiting high variability in the examined trait (CV > 30%) (Supplementary Table S4).

Shape stability differed markedly among the cultivars. While many accessions showed virtually no interannual variation, others, notably ‘Aprimira’ and ‘Herman’, exhibited higher variability. Nevertheless, fruit shape belonged to the most stable characteristic, as only six out of the 36 evaluated varieties did not show low to negligible variability.

In the present study, the fruit skin color of most of the evaluated cultivars (58%), including the control cultivars ‘Stanley’ (c) and ‘Cacanska Lepotica’ (c), was characterized by a coefficient of variation of less than 10%. In 39% of the evaluated cultivars, this parameter ranged from 10% to 20%. Only one cultivar, ‘Mirabelka Metska’, exhibited pronounced year-to-year fluctuations in fruit color, with the coefficient of variation exceeding 51.2%. Cultivars identified as donors of stable blue skin color (coefficient of variation ≤ 10%) include ‘Ortenauer’, ‘Hamanova svestka’, ‘Valor’, ‘Topgigant’, ‘Tuleu Gras’, ‘Dambovita’, ‘Carpatin’, ‘Belgicka modra’, ‘Toptaste’, ‘Svestka domaci’, ‘Chodovlicka’, ‘Herman’, and ‘Vankova Chrudimska’. These cultivars exhibit blue skin color or its variations with red and purple hues.

Similarly, flesh color fluctuated frequently among years and several varieties such as ‘Stanley’ (c), ‘Bonne de Bry’, ‘Cacanska rodna’, ‘Herman’, ‘Schueleho rana’, ‘Topgigant’, ‘Topking’, and ‘Valor’ showed very high interannual variability exceeding 30%.

Wax bloom was among the most stable characteristics, with interannual fluctuation higher than 30% observed in only a single cultivar, ‘Aprimira’.

A high degree of variability in the bruising resistance trait exceeding 30% was observed in the cultivars ‘Excalibur’, ‘Mirabelka Flotova’, and ‘Mirabelka Metska’. In contrast, cultivars such as ‘Cacanska Lepotica’ (c), ‘Anna Spaeth’, ‘Cacanska rodna’, ‘Bystricka muskatova’, ‘Carpatin’, ‘Chodovlicka’, ‘Vankova Chrudimska’, ‘Hamanova svestka’, ‘Herman’, ‘Opal’, ‘Schueleho rana’, ‘Svestka domaci’, ‘Timocanka’, ‘Topstar’, ‘Toptaste’, ‘Tuleu Gras’, ‘Valjevka’, ‘Bellamira’, and ‘Mirabelka Nancy’ exhibited low variability of this trait (10% or less).

Both skin firmness and flesh separability showed relatively high variability. The cultivars ‘Stanley’ (c), ‘Bonne de Bry’, ‘Cacanska rodna’, and ‘Schueleho rana’ fluctuated considerably across the five evaluated years, with CV values exceeding 30%.

Regarding flesh firmness, CVs did not exceed 30% in any of the evaluated cultivars. In 53% of the cultivars, the CV ranged between 10 and 30%, while the remaining cultivars exhibited high stability of this trait (CV < 10%). This group included the cultivars: ‘Cacanska Lepotica (c)’, ‘Anna Spaeth’, ‘Mirabelka Nancy’, ‘Mirabelka Jaune de Plovdiv’, ‘Dambovita’, ‘Mirabelka Flotova’, ‘Hamanova svestka’, ‘Toptaste’, ‘Svestka domaci’, ‘Bellamira’, ‘Gabrovska’, ‘Topstar’, ‘Valjevka’, ‘Topgigant’, ‘Tuleu Gras’, ‘Schueleho rana’, and ‘Carpatin’.

Interestingly, the evaluated cultivars exhibited a nearly identical pattern of variability for both flesh texture and stone separability. The highest coefficients of variation were recorded in ‘Stanley’ (c), ‘Cacanska rodna’, and ‘Schueleho rana’. In the stone separability parameter, the cultivar ‘Timocanka’ also exhibited high variability of evaluation. All organoleptic traits (orthonasal aroma of the flesh, taste of skin, juiciness, taste of fruit, retronasal aroma, and perceived acidity/sweetness balance) showed an almost identical grouping with respect to variability. This trait instability was observed mainly in ‘Stanley’ (c), ‘Cacanska rodna’, and ‘Schueleho rana’.

According to the fruit weight, the most stable varieties were ‘Cacanska Lepotica’ (c), ‘Stanley’ (c), ‘Anna Spaeth’, ‘Cacanska rodna’, ‘Hamanova svestka’, ‘Opal’, ‘Bellamira’, ‘Katalonsky spendlik’, and ‘Mirabelka Nancy’. On the other hand, ‘Aprimira’, ‘Dambovita’, ‘Excalibur’, ‘Esslingenska svestka’, ‘Timocanka’, ‘Top 2000’, ‘Topgigant’, and ‘Toptaste’ belonged to the most variable cultivars.

All dimensional characteristics behaved similarly; ‘Dambovița’, ‘Esslingenska svestka’, ‘Mirabelka Flotova’, ‘Mirabelka Metska’, ‘Timocanka’, ‘Topgigant’, and ‘Topstar’ produced fruits with the largest size differences among the evaluated years.

Pedicel length likewise did not rank among the most stable traits, with most cultivars displaying moderate to high variability. Notably, all cultivars from the German ‘TOP’ series (among others) showed coefficients of variation above 30%. Only ‘Topking’ was more stable, with a CV range of 10–30%.

In nearly all cultivars, soluble solids content exhibited only moderate year-to-year fluctuations, with no accession exceeding a CV value of 30%. The highest SSC stability was observed in ‘Aprimira’, ‘Mirabelka Flotova’, ‘Svestka domaci’, and ‘Mirabelka Nancy’.

### 2.4. Correlation Between Traits

Correlation between individual characteristics was evaluated as Pearson’s correlation coefficients; values higher than 0.7 were considered as statistically significant and commented (Table 6).

A strong positive correlation was detected between fruit color and wax bloom intensity (r = 0.9). Among the internal organoleptic pomological characteristics, the strongest relationship was found between the taste of the fruit and retronasal aroma intensity (r = 0.8). As expected, strong positive correlation was also detected between taste of fruit and perceived acidity/sweetness (0.7). Analysis of the correlation matrix revealed that the strongest statistically significant associations occurred among traits describing fruit size, specifically weight, height, width, and thickness. There were strong positive correlations between fruit weight and fruit height (r = 0.8), width (r = 0.9), and thickness (r = 0.9). Similarly, fruit height showed high correlations with fruit width (r = 0.8) and thickness (r = 0.8) but the highest correlation was found between fruit width and thickness (r = 1.0).

### 2.5. Comparison of Genetic Groups from a Phenotypic Perspective

We also attempted to compare the genetic groups defined by the dendrogram in order to identify the key phenotypic characteristics that distinguish the individual clusters. The main limitation of the statistical evaluation was the markedly uneven number of cultivars in each group. The Blue group of modern varieties included 12 cultivars; the Red group of landraces and Balkan accessions 17 cultivars; the Green group of gages, mirabelles and related types five; and the Orange group, the smallest one, only two. By analyzing cultivar-level means, each cultivar was treated as an independent observational unit. Group differences were evaluated using permutation-based ANOVA, which provides a distribution-free and assumption-robust comparison suitable for strongly unbalanced group sizes. However, the power of permutation tests is inherently limited when one group contains only two independent observations, which restricts the ability to detect statistically significant differences even when numerical contrasts are large. Therefore, mean values for all groups and for each trait were also provided.

Permutation-based ANOVA identified significant differences in ten evaluated traits (Table 7). The most pronounced contrasts were observed in fruit weight and dimensions, followed by fruit shape, color and bruising resistance. Since this test does not indicate which specific groups differ, a subsequent pairwise permutation test was applied to directly compare the genetic groups (Table 8).

The most significant differences were observed in size parameters. In most cases these differences were highly significant, with the Blue group clearly dominating over the Red, Green and Orange groups. The Green and Orange groups had significantly different fruit shape than the Blue and Red groups, with gages and mirabelles being spherical and plums being oval. These groups also differed in fruit color, concomitantly with wax bloom. Gages and mirabelles were usually yellow. However, plums from the Blue and Red groups ripened into dark blue-violet colors. The Green group demonstrated significantly lower bruising resistance than the other varieties. Mirabelles from the Orange group were the poorest ones in the parameters taste of fruit and retronasal aroma of fruit. Interestingly, the modern varieties (the Blue group) showed higher skin separability values than the landraces and Balkan varieties (the Red group). The Orange group stood out in parameter soluble solids content. However, due to the group containing only two varieties, the results were not significant.

### 2.6. Hierarchical Clustering Based on Phenotypic Traits and Comparison with Genetic Analyses

Phenotypic evaluation of all 22 traits was performed and the results were used to construct a phenotypic dendrogram. Then the phenotypic dendrogram was compared with the genetic one. Additionally, we examined which traits were most strongly influenced by the underlying genetic structure, despite the fact that the SSR markers used for genotyping were not associated with any pomological characteristics.

**Figure 7:**
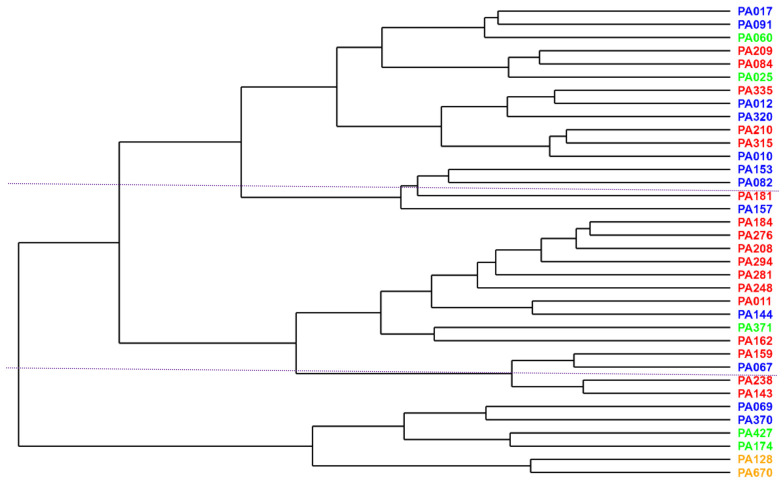
The dendrogram created from the phenotypic data of all 22 evaluated traits using ANOVA. The violet dotted lines separate individual branches. The codes of the individuals correspond to Table 1. The code colors correspond to Figure 2.

Hierarchical agglomerative clustering using Ward’s minimum variance method was also performed on the entire collection of 36 plum cultivars considering all 22 evaluated traits. The resulting dendrogram (Figure 7) was separated into three main branches. Codes of cultivars were colored according to genetic groups to enable the comparison of genetic and phenotypic data. A pairwise permutation test (Table 9) identified significant phenotypic differences distinguishing the branches.

The Upper branch is mainly composed of varieties from the Blue genetic group. Several cultivars from the Red group are also present, as well as ‘Opal’ (PA060) and ‘Bonne de Bry’ (PA025), which belong to the Green group. No universal traits were identified for assigning them to this branch. Overall, plums from this branch are significantly larger and juicier than those from the other two branches.

In the Middle branch, varieties from the Red group of landraces and Balkan and Romanian cultivars clearly prevail, along with ‘Cacanska Lepotica’ (c) (PA067) and ‘Topstar’ (PA144) from the Blue group and ‘Bellamira’ (PA371) from the Green group. These three varieties were assigned to this branch not for one reason, but rather for a combination of more traits that are responsible for their positions in the dendrogram. Interestingly, the phenotypic evaluations of individual traits of these varieties are usually near the minimum or maximum of this group. Fruit size parameters are the most statistically different traits. The Upper branch represents cultivars with bigger fruits and the Lower branch represents varieties with smaller fruits. Plums from the Middle branch are also characterized by higher flesh firmness values and a more prolonged fruit shape.

The Lower branch contains two out of five varieties from the Green group of gages and mirabelles, both varieties from the Orange group of mirabelles and two cultivars from the Blue group (PA069—‘Katalonsky spendlik’ with unknown pedigree; and PA370—‘Aprimira’, a sibling of ‘Mirabelle von Herrenhausen’). Although ‘Katalonsky spendlik’ and ‘Aprimira’ are larger than the other cultivars in this group, their color and wax bloom are probably the main traits that led to their assignment to this group. This branch differs from the other two by having statistically significantly smaller size parameters, fruit color (usually yellow), and low wax bloom, as well as a more spherical shape.

When the results of hierarchical agglomerative clustering using all 22 traits (Figure 7) are compared with the genotyping data (Figure 2), both dendrograms show some similarities. Two mirabelles from the Orange group form a separate subbranch also within the Lower branch of the phenotypic dendrogram, mirroring the situation in the genetic dendrogram. In this case, ‘Mirabelka Metska’ and ‘Mirabelka Nancy’ were separated due to the DAPC results. Most varieties from the Red genetic group form a compact cluster from a phenotypic perspective as well (the Middle branch), whereas nearly all the Blue group cultivars are localized in the Upper branch of the phenotypic dendrogram. Thus, the Green group of greengages and mirabelles represents the phenotypically least conserved genetic group, and its members may be found in different branches. However, this group also contains hybrids of mirabelles and large-fruited, blue-colored plums, which predispose them to be included in different phenotypic groups.

**Figure 8:**
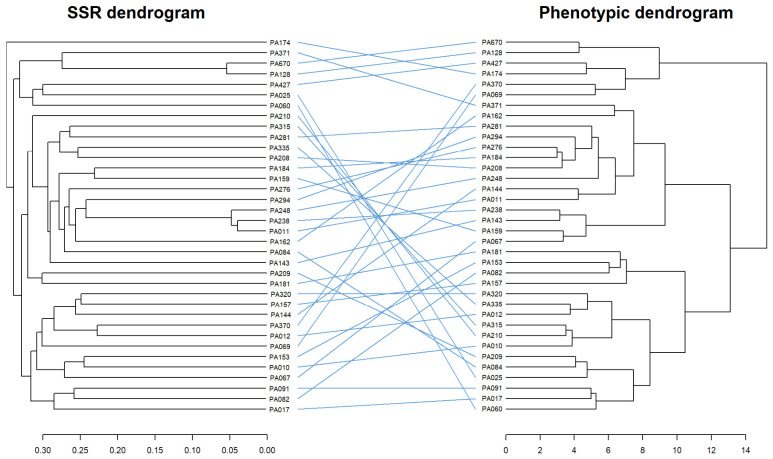
The tanglegram created using genotypic data from 12 SSR markers and phenotypic data from all 22 evaluated traits. The blue lines show varieties that are distributed in exactly the same branches in both dendrograms. The codes of the individuals correspond to Table 1.

However, the construction of the tanglegram (Figure 8) revealed only partial agreement between the two dendrograms. Baker’s gamma coefficient (0.37) indicates a weak to moderate correspondence in the ordering of sample pairs. This suggests that only some cultivar groupings are shared across both datasets. The cophenetic correlation coefficient (0.30) further shows low similarity in the pairwise distances, meaning that genetic relatedness does not closely reflect phenotypic similarity. The entanglement value (0.60) confirms a moderate level of structural mismatch between the two dendrograms. Mantel’s test revealed a weak yet statistically significant correlation between phenotypic and SSR-based genetic distances (r = 0.152, *p* = 0.018).

**Figure 9:**
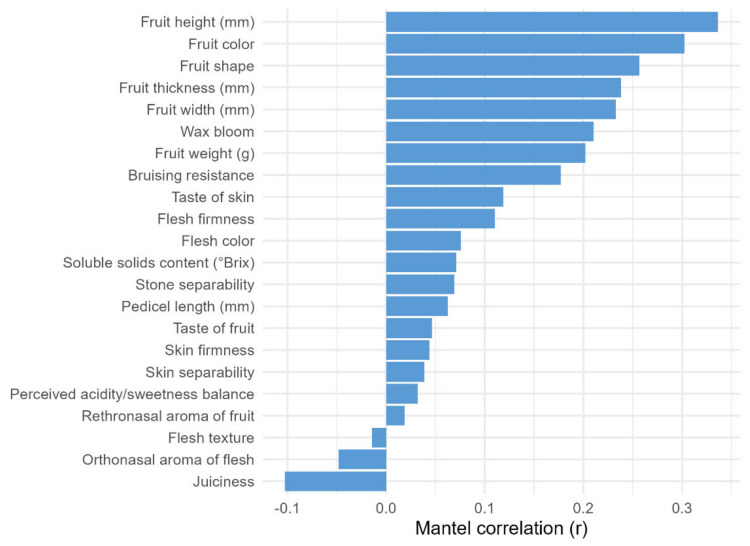
The Mantel’s correlations between the SSR-based genetic distance matrix and 22 individual phenotypic traits.

The low to moderate similarity indicated by the tanglegram can be explained by the Mantel’s correlations between the SSR-based genetic distance matrix and the individual phenotypic traits. These correlations revealed substantial differences in the degree of genetic determination among the traits (Figure 9). The highest correlations (r > 0.25) were observed for three fruit traits only: fruit height (r = 0.34), fruit color (r = 0.30), and fruit shape (r = 0.26). Relatively strong associations with genetic structure (r = 0.20–0.25) were detected for four additional traits, namely fruit thickness (r = 0.24), fruit width (r = 0.23), wax bloom (r = 0.21), and fruit weight (r = 0.20). However, the remaining 15 traits displayed correlations below 0.20, with several even showing negative values.

## 3. Discussion

Our study involved the long-term evaluation of 22 pomological traits in 36 genetically diverse cultivars of hexaploid European plum. The aim was to provide breeders with a foundation for establishing a core breeding collection of parents that reliably express specific, horticulturally valuable traits for both processing cultivars and table plums.

### 3.1. Genetic Analyses

First, the cultivars were genetically analyzed using 12 SSR markers [5] to determine their relationships and to assess the genetic heterogeneity of the selected collection. Several clonally derived cultivars that are commonly distinguished in practice were also included as controls.

As a first step, an analysis of the parental combinations of the selected cultivars was performed. This approach is not often described in the literature, but it can be carried out [19,26] using the POLYGENE software, for example, which can handle polyploid samples [21]. This analysis confirmed several known parentage combinations, verified presumed clonality, and identified new parental relationships. However, some reported parentages could not be confirmed, as in the case of the cultivar ‘Cacanska lepotica’. As Decroocq et al. previously noted [19], the published parentage of this cultivar is incorrect. Thus, our results independently confirm an alternative parental combination and suggest the likely true parents of this widely propagated plum. Due to the very limited number of publications on parentage combinations (e.g., [19,26]), this study, which searched for the parents of 36 cultivars within a database of 242 unique genotypes [5], substantially expands of the genetic verification of published parentage records.

However, it should be kept in mind that this parentage analysis is limited by the set of potential parents included in the reference collection. There are several possible factors that can explain why some known parentages could not be confirmed: (1) pollination of the mother tree by unintended pollen; (2) replacement at various levels (parents, offspring, or germplasm used for verification—a risk minimized here by testing many cultivars from multiple sources); (3) the possibility that the parent trees used for crossing were clones differing in two or more alleles from the analyzed samples of the same cultivars in the RBIP Holovousy germplasm collection—only parent combinations differing by a single allele were accepted to reduce bias; and (4) laboratory handling errors—although these can largely be excluded because all samples were analyzed in biological duplicates with identical results. Since the literature on parentage verification is very limited, future studies by independent laboratories are needed to confirm the suggested parental combinations. Nevertheless, the parentage analysis confirmed the broad genetic origin of the evaluated collection, which was the main objective of these tests.

Genetic diversity was further assessed using several complementary approaches. Principal Component Analysis (PCA) revealed that PC1 and PC2 explained only 10.6% and 10.2% of the total variance, respectively. These low proportions are typical of SSR-based analyses of polyploid species, as also demonstrated for hexaploid *Prunus domestica* by Makovics et al. [15]. Therefore, PCA was supplemented with Discriminant Analysis of Principal Components (DAPC) to maximize variation between groups. Automated selection of the optimal number of clusters using the Bayesian Information Criterion found K = 1, which suggests weak genetic structure within the dataset. This result reflects the high genetic diversity of the selected cultivars and supports their suitability for establishing a genetically diverse core collection. To further explore the potential substructure of the selected cultivar collection, K values from 3 to 5 were tested manually. A comparison of DAPC clustering with the dendrogram based on SSR markers revealed that the division into four subpopulations was the most biologically meaningful, which is why it was used throughout the manuscript. Notably, separating classical European plums and semi-plums into two genetically distinct groups (the Blue and Red ones) may be valuable for breeders aiming to broaden the relatively narrow genetic base of hexaploid plums, as Zhebentyayeva et al. previously documented [13].

Genetic diversity was further evaluated using Bruvo’s distance. However, this method does not have universal thresholds for interpreting null, slight, or strong genetic diversity because the values depend heavily on the SSR markers used and the population analyzed. For example, this is illustrated by Manco et al. [16], who used five EST-derived SSRs (EST-SSRs) and five genomic SSRs (gSSRs) to analyze the same collection of 44 hexaploid European plums. While the EST-SSRs produced Bruvo’s distances up to 0.6, the gSSRs yielded substantially higher values (up to 0.925). Urresterazu et al. [14] reported Bruvo’s distances for 135 unique hexaploid plum cultivars genotyped with 11 SSR markers. Several pairs differed by less than 0.05, corresponding to clonal relationships. STRUCTURE-defined groups showed mean distances ranging from 0.30 ± 0.15 to 0.58 ± 0.08. The clonality threshold in this study was set at 0.05, which corresponds to the clonal value in Urresterazu et al. [14]. All other non-clonal cultivar pairs showed Bruvo’s distances greater than 0.297. Thus, clonal pairs were clearly separated from all other cultivar pairs. The results of the parentage analysis helped establish a threshold to distinguish between related and unrelated cultivar pairs (0.45). Over 70% of the cultivar pairs in our dataset were classified as unrelated, which confirms the high genetic diversity of the selected collection.

Taken together, the genetic analyses confirmed that the selected cultivars represent a highly diverse germplasm set, fulfilling the essential requirement for establishing a core collection according to Brown (1989) [27].

### 3.2. Phenotype Analyses

Detailed pomological evaluation was conducted over five consecutive years to minimize interannual variation and to assess phenotypic stability, because cultivars that consistently express desirable traits are particularly valuable for breeding. A total of 22 phenotypic traits representing a broad repertoire of characteristics were investigated to select parents for breeding new varieties intended for direct consumption or industrial processing as these two uses may require different desired traits. Consumer acceptance of European plum cultivars depends on a combination of external pomological traits and sensory attributes. These include fruit size and shape, skin color intensity and uniformity, and overall market appearance. Key eating-quality parameters, such as balanced sweetness, aroma, juiciness, and flesh firmness, also play a role [10,28,29,30,31,32,33]. On the other hand, European plum fruits are also an important raw material for the processing industry, and dried plums are among the most common processed products. To produce high-quality dried plums, the fruit should be a dark blue to dark purple color, have firm skin and flesh, a good taste, a smaller size and stones that can easily be separated from the flesh [34]. Plum for jam production should have high natural sugar levels, sufficient pectin, balanced acidity, and a rich aromatic profile. They should also have soft, easily cookable flesh to ensure thick, flavorful, naturally sweet preserves. Finally, plums suitable for distillation should have high fermentable sugar content, balanced acidity, and strong aromatic precursors. They should also have easily separable pits and soft, degradable flesh to ensure efficient fermentation and a clean, aromatic spirit. This implies that the requirements for parents vary according to the ultimate goal of breeding [35].

Among external pomological characteristics, skin color is one of the most attractive traits for consumers. Cultivars with green skin and its variations are particularly preferred in France and in some regions of Germany. In most other European countries, however, there is a predominant demand for fruits with blue skin color [30]. It should be noted that the final fruit skin color is also influenced by external environmental factors, especially the growing site, temperature regime, canopy training, light distribution, and the overall set of ecological conditions characteristic of a given production region [36]. For example, studies conducted on certain sweet cherry cultivars have demonstrated that fruit color variation depends on both cultivar-specific traits and environmental influences [37]. The plum cultivar collection in this study may be valuable material for the breeding of new cultivars in various countries, depending on consumer preferences regarding fruit color in a given region. ‘Aprimira’, ‘Bellamira’, ‘Katalonsky spendlik’, mirabelles or ‘Excalibur’ may be used to produce cultivars with lighter-colored fruits. On the other hand, ‘Cacanska lepotica’, ‘Stanley’, ‘Carpatin’, ‘Dambovita’, ‘Hamanova svestka’, ‘Ortenauer’, ‘Topgigant’, ‘Toptaste’, ‘Tuleu Gras’, ‘Valor’ or clones of ‘Svestka domaci’ can be used to breed new varieties with dark blue fruits. The blue-to-violet color of plums is determined by anthocyanins [38]. The synthesis of these antioxidants is regulated by MYB transcription factors that present many different genetic variants responsible for the fruit color as clearly demonstrated by Fiol et al., 2021 [39] in the closely related Japanese plum (*Prunus salicina* Lindl.). The blue color is usually dominant. However, the color of the fruit in the parents is not always fully predictive of the fruit color in the siblings, and the F1 generation may exhibit a variety of fruit colors [39].

The bruising resistance of fruits is very important phenotypic trait because higher resistance prevents or diminishes fruit damage. Therefore, enhanced bruising resistance is a trait selected for in both modern and traditional cultivated cultivars, as it has broader market relevance [25,40,41]. Nearly all of the evaluated cultivars were concentrated in the middle to upper part of the scale for this trait (6.0–7.0 points), which suggest the suitability of these varieties as parents in breeding programs. The cultivars with the highest bruising resistance were ‘Hamanova svestka’ and ‘Valjevka’. Bruising resistance in stone fruits is a very complex trait that may be influenced by many factors, some of which are variety-independent, such as for example harvest methods (mechanical harvesting, hand picking), maturity at the time of harvest, and harvest time during the day [25]. However, other factors are variety-dependent, including skin and flesh firmness together with elasticity, and cell wall strength, pectin composition, and softening enzymes activity [42]. Due to the high complexity of this trait, a careful selection of parents for breeding is crucial.

Flesh firmness in *Prunus* species (such as peaches, cherries, apricots, and plums) is a polygenic trait governed by complex genetic architecture and coordinated cell wall modification pathways [43]. It is such an important trait that molecular markers suitable for molecular marker-assisted selection (MAS) of seedlings during breeding were developed in diploid *Prunus* species [44,45,46]. In our collection, the most commercially important cultivars were concentrated in the middle to upper part of the scale (5.5–6.5 points). This suggests that medium to high flesh firmness is a typical trait of cultivars with broader practical applicability. To increase flesh firmness in progeny, ‘Gabrovska’ is the best parent with a flesh firmness score of 7.4. On the other hand, the parents with low flesh firmness include ‘Esslingenska svestka’, ‘Mirabelka Flotova’, and ‘Vankova Chrudimska’ (less than 5 points).

Stone separability is a key technological trait of European plums, particularly for cultivars intended for processing. The easy detachment of the stone from the flesh makes industrial processing more efficient and directly affects processing yield and product integrity. Additionally, consumers prefer fruits with free stones. The separability of the stone from the flesh is a genetically determined trait as is evident from the closely related peach (*Prunus persica* L.). The freestone vs. clingstone phenotype is correlated with the melting-flesh vs. non-melting-flesh phenotype. Both are controlled by a single locus containing at least one endopolygalacturonase (endoPG) gene. Allelic variation at this locus explains differences in stone adhesion and fruit softening [47]. The genetic background of the freestone phenotype has also been confirmed by others [48,49], so great attention should be paid to selecting appropriate variety with good stone separability. In our study, the varieties with the highest core of 9.0 are ‘Bellamira’, ‘Bystricka muskatova’, ‘Gabrovska’, ‘Hamanova svestka’, ‘Opal’, and ‘Topstar’. The cultivars with the lowest scores bellow 6 points are ‘Anna Spaeth’, ‘Mirabelka Flotova’, and ‘Timocanka’ (whose flesh partly remains on the stone).

Taste is a highly complex key indicator of fruit quality, because it fundamentally determines its attractiveness to consumers. Taste is shaped by the balance of major components such as sugars, organic acids, tannins, vitamins, and aromatic compounds. Taste also exhibits considerable variability depending on cultivar origin. Perceived sweetness is the dominant sensory signal that significantly influences the overall quality evaluation. Consumers in southern Europe and Asia generally prefer fruit with a distinctly sweet flavor profile. However, in many other regions a balanced combination of sweetness and acidity is more highly valued [50,51]. For fresh consumption, taste and aroma are the main criteria for cultivar selection [52]. Therefore, cultivars that achieved 7–9 points in our long-term sensory evaluation can be recommended, namely: ‘Anna Spaeth’, ‘Aprimira’, ‘Bellamira’, ‘Carpatin’, ‘Dambovița’, ‘Esslingenska svestka’, ‘Hamanova svestka’, ‘Vankova Chrudimska’, ‘Katalonsky spendlik’, ‘Opal’, ‘Topking’, ‘Toptaste’, ‘Tuleu Gras’, and ‘Valjevka’. These may expand the portfolio of recommended good- and very good-tasting varieties recommended in Butac M. (2020): ‘Tuleu gras,’ ‘Centenar,’ ‘Gras ameliorat,’ ‘Grase de Becs,’ ‘Uriașe de Sibiu,’ ‘Agent,’ and ‘Andreea’ in Romania; ‘Bijelica sitna’ and ‘Prskulja’ in Bosnia and Herzegovina; ‘Moravka’, ‘Metlaš’, ‘Obični piskavac,’ and ‘Čačanska Najbolja’ in Serbia; ‘Auerbacher,’ ‘Ortenauer,’ and ‘Wangenheims’ in Germany; ‘Italian prune’ and ‘President’ in the USA; and ‘d‘Agen’ in California and France. From a genetics standpoint, several molecular markers have been developed for different taste-determining molecules [53]. However, fruit taste is such a complex trait that it is not possible to perform MAS on seedlings with good overall taste-potential even in diploid *Prunus* species. Thus, great care must be taken in selecting parents for the breeding of new cultivars with tasty fruit. One selection criterion may be high soluble solids content (SSC). According to Neumüller (2011) [35], this parameter ranges from 12 to 32 °Brix in European plum genotypes. A value over 12.0 °Brix SSC is considered the minimum threshold for consumer acceptance [54,55], and thus represents a key criterion for evaluating plum fruit quality. All cultivars evaluated in our study exceeded this threshold. The highest °Brix values were observed in central European conditions in the cultivars ‘Topking’, ‘Anna Spaeth’, ‘Aprimira’, and ‘Toptaste’ (≥22.0 °Brix).

Today, fruit weight and size are key parameters when monitoring the fresh fruit market. Bigger fruits are more highly valued and bring a higher profit to farmers. Based on this criterion, the cultivars in our collection with the highest average fruit weight can be identified: ‘Carpatin’, ‘Timocanka’, ‘Topgigant’, ‘Topstar’, ‘Toptaste’, ‘Valor’, ‘Anna Spaeth’, and ‘Excalibur’, with average fruit weight exceeding 45 g. Fruit weight is a genetically determined quantitative trait [56]. However, improving this trait in hexaploid plums is difficult. Only a few hybrids surpass their parental forms in this trait [57]. According to the literature, if the cultivar ‘Stanley’ is one of the parental components, its hybrid progeny usually exhibits higher fruit weight [58,59]. However, in our study, the average fruit weight of this cultivar was only 37.1 g, corresponding to the medium weight category.

### 3.3. Variability of Traits Across Years

The variability parameter of traits indicates to breeders the reliability with which desired characteristics can be transmitted to progeny when developing new cultivars. The variability of traits across years usually does not exceed 30% as expressed by the coefficient of variation, which is indicative of the suitability of the described collection as parents for breeding. The most variable traits were size parameters. However, the cultivars were grown in a non-irrigated orchard and fluctuation in weight and size can be expected.

### 3.4. Correlation Between Traits

Correlation analysis of pomological traits is an important tool for evaluating and breeding fruit crops, because it identifies relationships among indicators of fruit quality, productivity, and technological suitability. Determining positive or negative correlations between traits allows for the indirect selection of promising genotypes for breeding. A strong (Pearson’s correlation coefficient 0.71–0.9) or very strong (greater than 0.9) correlation was observed between traits in some trait combinations.

A strong positive correlation was detected between fruit color and wax bloom intensity (r = 0.9). As previously reported [60], the visual perception of wax bloom on plum fruit differs markedly between yellow-skinned and blue/purple-skinned cultivars due to the interaction between the epicuticular wax microstructure and the optical properties of the underlying skin. The epicuticular wax on *Prunus domestica* forms crystalline platelets that diffusely scatter light, producing the characteristic bluish-white bloom. On dark blue or purple fruit, this scattered light contrasts strongly with the low-reflectance background, making the wax layer appear thicker and more visually prominent. After wax removal, the blue fruit color of many varieties may shift toward violet or very dark blue. In contrast, the skins of yellow fruit exhibit higher intrinsic reflectance in the visible spectrum, reducing the contrast between the wax bloom and the underlying epidermis. Consequently, the same wax load appears less intense or even inconspicuous. Wax removal in green/yellow plums produces almost no visible color change [61]. Therefore, these traits exhibited strong dependency, but the correlation is not genetically determined. Rather, it is influenced by the visual perception of wax bloom on different backgrounds.

In fact, the wax bloom on plums is not directly affected by the thickness of the wax layer. Waxes on the surface of fruits are typically evaluated indirectly by measuring surface luster, a reliable, non-destructive indicator of wax coverage [60]. According to luster measurements, plum type (blue European plum, yellow mirabelle, and greengage) has only a negligible influence on the overall range of luster values, as reported by Zemke et al. [62]. Similar results were obtained by Mukhtar et al. [60] showing that luster values varied slightly among cultivars. Mirabelle and greengage had lower luster levels than blue-colored European plums corresponding to higher amounts of surface wax per unit area. Therefore, it is important to distinguish between the visual perception of wax bloom and the actual thickness of the wax layer. Visual perception is important from a consumer standpoint because mechanical damage to the wax layer is far more noticeable on blue fruit than on yellow or green fruit. This indicates rough handling of the fruit that can shorten its shelf life. The true thickness of the wax layer is a valuable trait for postharvest variety performance because it reduces fruit water loss, increases their resistance to mechanical or microbial damage and prolongs fruit shelf life [60,63]. However, the wax content on the plum surface has not yet been measured.

Among the internal organoleptic pomological characteristics, the strongest relationship was found between the taste of the fruit and retronasal aroma intensity (r = 0.8). This highlights the fundamental role of volatile aromatic compounds (VOCs) in creating a complex flavor profile. Similarly, a strong positive correlation was detected between the taste of the fruit and the perceived acidity/sweetness (0.7). Fruit taste (e.g., sweetness and acidity) and retronasal aroma (i.e., VOCs released during chewing) are closely related because VOCs interact with sugars and acids to create the overall perception of flavor. For example, Baldwin et al. [64] demonstrated that the level of aroma compounds affected the perception of sweetness and sourness in tomatoes. In strawberries, both sweetness- and acidity-enhancing or -reducing VOCs have been identified [65].

Additionally, it should be noted that the fruit size parameters of weight, height, width, and thickness exhibited significant correlation relationships in the range of 0.8 to 1.0. These findings suggest a high degree of morphological synchronization of growth processes, meaning an increase in one dimensional parameter is accompanied by proportional increases in the others. Multiple studies demonstrate that fruit weight and fruit dimensions are strongly genetically and jointly inherited in horticultural crops. These traits typically share overlapping quantitative trait loci, indicating that the same genomic regions influence several aspects of fruit size simultaneously. For example, genome-wide association studies in tomatoes identified molecular markers associated with multiple fruit traits, including fruit weight, width, and height, indicating the pleiotropic effects of certain loci [66]. In the closely plum-related sweet cherry, fruit weight and size parameters were also highly correlated, and several markers were associated with different size traits or colocalized within a single haplotype block [45]. In peach, a model organism within the genus *Prunus*, major quantitative loci associated with fruit size and shape traits also colocalized in a genome-wide association study performed on a non-flat peach collection [67].

### 3.5. Genotype–Phenotype Associations

Finally, the genetic data were integrated with the phenotypic dataset. Two complementary approaches were applied. In the first approach, genetically defined groups served as a framework to examine phenotypic traits in order to identify the characteristics that distinguish genetic clusters. The second approach used phenotypes as the starting point, and identified traits that showed the strongest correlations with the allelic composition of the 12 SSR genotyping markers. Additionally, dendrograms constructed from genetic and phenotypic data were compared to assess the correspondence between the two sources of variation. To statistically evaluate differences in individual phenotypic traits between genetic groups, permutation-based ANOVA followed by permutation-based pairwise tests was used. This approach was selected because the dataset consisted of strongly unbalanced groups and non-independent repeated measurements collected over five years—conditions under which classical parametric tests violate key assumptions. Analyzing cultivar-level means rather than annual measurements, each cultivar was treated as an independent observational unit. This avoided pseudoreplication and reflected the biological reality that cultivars, rather than yearly observations, represent the true entities being compared. This method provided a robust, distribution-free assessment of group differences while maintaining statistical validity despite unequal group sizes. However, the presence of only two cultivars in the Orange group substantially limited statistical power. Although some traits showed large numerical differences between group means, the tests did not reach statistical significance. Therefore, the results should be interpreted also in terms of effect sizes and descriptive patterns, not just by a strict significance. Nevertheless, the analyses revealed several notable differences among the genetic groups. As expected, there were marked differences in traits such as fruit dimensions, weight, shape, color, and wax bloom intensity between groups, reflecting the traditional pomological classification of different plum types [13]. To point out the main features of individual groups, the Red group (landraces and Balkan varieties) had smaller fruit than plums in the Blue group and demonstrated lower skin separability than the modern Blue group cultivars. Gages and mirabelles (the Green group) had lower bruising resistance than all the other evaluated varieties. The French mirabelles from Nancy and Metz have a lower retronasal aroma and taste of fruit, but a higher soluble solids content.

A dendrogram was generated using hierarchical agglomerative clustering and Ward’s minimum variance method based on the mean values of all 22 evaluated phenotypic traits at the cultivar level. The genetic and phenotypic dendrograms showed several similarities. However, the genetically defined Green group of gages and mirabelles was dispersed throughout the phenotypic dendrogram. This segregation may reflect the parentage of some cultivars in the Green group, as some varieties (such as ‘Opal’ and ‘Bellamira’) are from crosses between gages or mirabelles and other pomological plum groups. In contrast, the Blue and Red groups remained largely intact. Nevertheless, the tanglegram comparing the two dendrograms showed only low to moderate similarity. This is to be expected, as the SSR markers used for genotyping were not associated with any evaluated phenotypic trait, and only 12 loci were analyzed. This finding is consistent with previous studies in diverse crops. The correspondence between phenotypic and SSR-based clustering was also limited in other scientific works [68,69,70]. Despite the low to moderate similarity between the genetic and phenotypic dendrograms, Mantel’s correlations between the SSR-based genetic distance matrix and each of the 22 phenotypic traits identified the traits most strongly correlated with genetic structure. As expected, these included fruit size, shape, and color parameters, as well as the closely related trait of wax bloom intensity. These traits have a relatively simple genetic background and the molecular markers associated with their variation are frequently reported in the literature, not only for diploid *Prunus* species [45,67,71]. Unfortunately, the hexaploid nature of the European plum is a big complication for MAS realization and, to our knowledge, it is not applied in plum breeding. All the greater attention must therefore be paid to the selection of parents for crossing.

### 3.6. Recommendations for Plum Breeders

Based on a conducted multi-year pomological evaluation, the following cultivars were selected for breeding new table varieties: ‘Aprimira’, ‘Belgicka modra’, ‘Bellamira’, ‘Carpatin’, ‘Dambovita’, ‘Hamanova svestka’, ‘Chodovlicka’, ‘Katalonsky spendlik’, ‘Mirabelka Metska’, ‘Mirabelka Nancy’, ‘Opal’, ‘Timocanka’, ‘Tuleu Gras’, and ‘Vankova Chrudimska’. The genotypes have above-average sensory quality (scores 5–9) for all internal organoleptic pomological characteristics (Table 4).

An analysis of the morphological and technological characteristics of the cultivars included in this study indicates that the most suitable genotypes for processing are ‘Cacanska Lepotica’ (c), ‘Cacanska rodna’, ‘Dambovita’, ‘Gabrovska’, ‘Ortenauer’, ‘Topking’, ‘Toptaste’ and ‘Valjevka’. These cultivars showed high stone separability scores (6–9 points), dark fruit color, and firm skin and flesh (6–9 points) and fruit weight exceeding 25 g suggesting that they are well-suited for industrial use due to their favorable processing-related traits.

To facilitate the selection of suitable parents for targeted crossing, the Supplementary Table S5 includes an Excel table that summarizes all phenotypic evaluations, visualized using a color-scale. Hiding irrelevant columns allows the Table to display only the desired traits enabling, for example, the selection of a cultivar with excellent sensory attributes (such as ‘Hamanova svestka’) for crossing with a large-fruited genotype (e.g., ‘Topgigant’ or ‘Timocanka’) to obtain potentially improved progeny.

## 4. Materials and Methods

### 4.1. Experimental Site and Plant Material

The evaluation was carried out from 2019 to 2023 at the Department of Genetic Resources of the Research and Breeding Institute of Pomology Holovousy Ltd. (RBIP) in the Czech Republic. The plum cultivars assessed were part of the RBIP genebank, which was established in 2012 within the fruit germplasm collections located in Holovousy (eastern Bohemia), Jičín district of the Hradec Králové region (50°36′67″ N, 15°56′64″ E; 290 m a.s.l.). This area has a mean annual temperature of 8.4 °C and an annual precipitation of 663.5 mm. The orchard was planted in 2012 with a spacing of 6.0 × 5.0 m, with three trees representing each cultivar. All cultivars were grafted onto myrobalan (*P. cerasifera*) rootstock, and the plantation was not irrigated.

The trees were trained as quarter-standard trees with an open-center vase canopy and well-developed scaffold branches. The stem was 0.8–1.2 m high. Annual sanitary pruning was conducted in April–May to maintain tree size and canopy architecture. Dead, broken, or diseased branches were removed back to healthy wood. The herbicide strip under the tree rows was kept weed-free, and the alleyways were maintained as regularly mowed grass to limit erosion and facilitate orchard operations, and allow mechanization. Standard pest control, fertilization, and other routine agrotechnical measures were applied throughout the study period.

Fruit samples for pomological evaluation were collected at harvest maturity from the plum collection for each cultivar (names according to GRIN Czech). Two widely grown and well-characterized cultivars with contrasting ripening times were included as reference controls: ‘Stanley’ (c) (late-ripening) and ‘Cacanska Lepotica’ (c) (medium-early-ripening). These cultivars are widely distributed internationally, exhibit stable and well-described pomological traits, and are broadly suitable for both fresh consumption and processing.

### 4.2. Pomological Description

Pomological traits were assessed using the established nine-point descriptor scale for European plum. This scale is in accordance with the Test Guidelines of the International Union for the Protection of New Varieties of Plants (UPOV) for *Prunus domestica* L. The scale was adapted to reflect the specific diversity and composition of the evaluated plum cultivars [72]. The descriptor set and scoring procedure were approved for use within the national documentation framework and archived in the Czech GRIN (Germplasm Resources Information Network) information system for plant genetic resources.

The pomological description of fruits collected during the evaluation years comprised the following parameters:

- External pomological traits: fruit shape (1—spherical; 2—oval; 3—long oval; 4—long bulbous; 5—ellipt; 6—long prune; 7—ovoid; 8—drop-shaped; for fruit shape, the rating scale goes only up to 8); fruit color (1—green; 2—yellow; 3—yellow-red; 4—red; 5—red-purple; 6—blue; 7—red-blue; 8—blue-purple; 9—blue-brown to black); flesh color (1—whitish green-yellow; 3—green-yellow; 5—yellow; 7—yellow-orange; 9—orange); wax bloom (1—absent; 3—weak; 5—medium; 7—strong; 9—very strong); bruising resistance (1—very low; 3—weak; 5—medium; 7—strong; 9—very strong). Bruising was induced by gentle manual compression of the fruit using the evaluator’s fingers and subsequently assessed subjectively using a standardized rating scale.
- Internal structural pomological characteristics: skin firmness (1—tender; 5—medium; 9—firm); skin separability (1—very poor; 5—moderate; 9—very good); flesh firmness (1—very soft; 3—soft; 5—moderately firm; 7—firm; 9—very firm); flesh texture (1—fibrous and coarse; 3—less fibrous; 5—finely fibrous; 7—fine; 9—watery/loose); stone separability (1—very poor; 3—poor; 5—flesh partly remains on the stone; 7—slight flesh remains; 9—no flesh remains on the stone).
- Internal organoleptic pomological characteristics: orthonasal aroma of flesh (1—strongly unpleasant; 5—weak but pleasant; 9—intense and pleasant); taste of skin (1—astringent; 5—acidic/sour; 9—sweet); juiciness (1—rather dry; 5—juicy; 7—very juicy); taste of fruit (1—very poor to unacceptable; 3—weak and indistinct; 5—average/good; 7—very good; 9—excellent); retronasal aroma of fruit (1—absent; 3—barely perceptible; 5—perceptible; 7—pronounced; 9—very pronounced); perceived acidity/sweetness balance (1—acidic; 3—tart; 5—sweet–tart; 6—tart–sweet; 7—slightly sweet; 9—very sweet).
- Measurable pomological traits: fruit weight (g); fruit height, fruit width, and fruit thickness (mm); pedicel length (mm); soluble solids content (SSC) was expressed in °Brix using a digital hand refractometer (Bellingham + Stanley Ltd., model Brix 54, Tunbridge Wells, Kent UK).
- The intermediate (non-listed) scores between 1 and 9 represent ordinal mid-classes between the two nearest defined category values. To ensure the interannual consistency of qualitative trait evaluation (e.g., juiciness, aroma), all sensory assessments were conducted by the same trained evaluators using standardized UPOV descriptors, with regular standardization procedures and reference samples applied to minimize scoring variability across years.

### 4.3. Statistical Data Analysis

Statistical analyses of phenotypes were performed using Minitab 19 [73]. Mean values, standard deviation (SD), and coefficient of variation (CV) were calculated to evaluate data variability. Cultivars were classified according to CV as follows: low variability (CV ≤ 10%), moderate variability (CV = 10–30%), and high variability (CV > 30%).

### 4.4. Genetic Analyses

The SSR data used for the molecular genetic analyses were obtained from Čmejlová et al. [5] or generated using the methods described herein. The allelic compositions of twelve SSR markers were used for all analyses.

Parentage analysis was performed for all 36 described accessions against 242 unique genotypes in the 12 SSR genotyping system described in [5] using the Phenotype method in POLYGENE version V1.7 [21], searching for parent pairs with unknown sex and allowing selfing. All positive LOD parent candidates with a Trio locus mismatching score of 0 or 1 were extracted, and clones of the respective accessions were disregarded as potential parents.

For Principal Component Analysis (PCA), the genotypes were imported into R, version 2026.01.0, Build 392 [74] for downstream statistical processing. Missing allele values were imputed by replacing them with the mean value of the corresponding marker column. PCA was performed using the prcomp function with centering and scaling enabled, to allow visualization of the major axes of genetic variation. The first two principal components (PC1 and PC2) were extracted and combined with sample identifiers to generate a two-dimensional ordination dataset. The proportion of variance explained by each principal component was calculated from the squared standard deviations of the PCA loadings. For graphical representation, the ggplot2 package [75] together with ggrepel [76] was used to produce a scatterplot of PC1 versus PC2. Each point in the plot represents an individual genotype.

The dendrogram was constructed using Cavalli-Sforza and Edwards’ chord distance [77] and clustered using the Unweighted Pair Group Method with Arithmetic Mean (UPGMA) as originally described by Sokal and Michener [78], and implemented in the POLYGENE software version V1.7 [21].

For Discriminant Analysis of Principal Components (DAPC), the dataset was converted in R into a genind object using the adegenet 2.1.11 package [79,80], assuming hexaploidy and codominant SSR markers. Missing alleles were retained as NA during import and subsequently handled during PCA preprocessing. Prior to DAPC, PCA was performed to decorrelate allelic variables and reduce dimensionality, ensuring numerical stability and optimal performance of the discriminant analysis. The allele count matrix was extracted using tab and standardized using scale, with missing values imputed by column means. PCA was performed using the function dudi.pca from the ade4 package [81], retaining 50 principal components for subsequent clustering. Clustering of individuals was carried out using the k-means algorithm applied to PCA coordinates and 50 random starts. The resulting cluster assignments were used as prior groups for DAPC implemented in dapc (adegenet). The optimal number of PCA components retained in DAPC was determined using optim.a.score. DAPC scatterplots were generated using the scatter function, with cluster-specific colors and manually added point labels. The ggrepel package [76] supported non-overlapping label placement in supplementary visualizations. Since the script did not automatically choose the number of clusters due to the variability of the collection, the number of clusters was consecutively set to K = 3, 4, and 5 and the division of varieties into clusters was compared with the genetic dendrogram. Cluster membership probabilities were visualized using the compoplot function.

To calculate Bruvo’s distance in R, alleles were collapsed into a single multilocus field per marker (APF format) by concatenating all non-missing alleles for each individual. The resulting APF table was converted into a genind object from the adegenet package [79,80], assuming hexaploidy and codominant SSR markers. To account for potential clonality within the dataset, the object was subsequently transformed into a genclone object. Genetic distances among individuals were computed using Bruvo’s distance, which is designed specifically for microsatellite data in polyploid organisms and accounts for allele copy number and mutational stepwise differences. Bruvo’s distances were calculated using the function bruvo.dist from the poppr package [23], with a uniform repeat length of two base pairs for all loci. The resulting distance matrix was exported to an Excel file. To visualize genetic relationships, a heatmap of the Bruvo’s distance matrix was also generated using the pheatmap package [82], employing average linkage clustering. A histogram was also constructed in R.

A permutation-based one-way ANOVA implemented in R was used to identify differences among the predefined genetic groups. Phenotypic data for all 22 traits were averaged at the cultivar level prior to statistical evaluation. All analyses were conducted using the tidyverse package for data handling [75] and the coin package for non-parametric inference [83,84]. For each trait, a model of the form Trait ~ Group was tested using the oneway_test function with 9999 permutations. This provided permutation-based test statistics and *p*-values that do not rely on assumptions of normality or homogeneity of variances.

Pairwise permutation tests with Benjamini–Hochberg correction were performed using the rcompanion package of R [85]. Data handling and reshaping were carried out using the tidyverse collection of packages [75].

A phenotypic hierarchical agglomeration clustering analysis of 36 plum cultivars was performed in R using mean values of 22 quantitative traits at the cultivar level. Prior to the analysis, all traits were standardized to a zero mean and a unit variance to ensure equal weighting across variables with different measurement scales. A Euclidean distance matrix was computed from the standardized data, and hierarchical agglomerative clustering was carried out using Ward’s minimum variance method [86]. The resulting hierarchical tree was converted into a dendrogram object and visualized using the packages ggplot2 [75] and ggdendro [87].

Tanglegram was used to compare the dendrograms created from genetic and phenotypic data, respectively. SSR-based genetic relationships were imported into R as a Newick tree from POLYGENE (described above) and converted into a dendrogram using the ape package [88]. The phenotypic dendrogram stems from hierarchical clustering using Ward’s minimum variance method. Similarity was quantified using Baker’s gamma correlation [89], cophenetic correlation [90], and the entanglement coefficient [91].

Mantel’s test [92] evaluated the association between phenotypic and genetic distance matrices. Phenotypic variables of all 22 traits were imported into R, converted to appropriate data types, and used to compute pairwise phenotypic dissimilarities using Gower’s distance [93] with the daisy function from the cluster package [94]. For each SSR locus, allele frequencies were calculated and the resulting allele-frequency matrix was used to compute a genetic Gower’s distance. The association between the phenotypic and genetic distance matrices was evaluated using a Mantel’s test with Pearson correlation and 9999 permutations. This test was implemented in the vegan package to assess the significance of the correlation between phenotypic variation and SSR-based genetic structure. Similar Mantel’s tests were performed to assess the association between the SSR-based genetic distance matrix and each phenotypic trait individually.

## 5. Conclusions

This study shows that combining SSR-based genetic characterization with multi-year pomological phenotyping creates a solid foundation for evaluating European plum germplasm in the pre-breeding stage. Through the integration of parentage analysis, population-level genetic structuring, distance-based evaluation, trait stability assessment, and correlation analysis, the selected collection can be interpreted as both a set of individual cultivars and a structured genetic and phenotypic resource for targeted breeding. The results confirmed that considerable diversity remains available within cultivated *Prunus domestica* germplasm, including variation in fruit appearance, internal quality, technological traits, and sensory attributes. The results also showed that some traits are stable enough across years to be considered reliable selection targets. From a breeding perspective, this combined evaluation identified candidate donor parents with complementary trait profiles for developing fresh-market and processing-oriented cultivars, thereby reducing the uncertainty associated with parent choice in a long-generation perennial crop. Overall, this study supports the use of germplasm mining as a practical strategy to transform ex situ fruit genetic resources into usable breeding material. It also provides a methodological basis for future selection schemes that combine molecular identity, genetic relatedness, phenotypic performance, and trait stability under growing conditions.

## Figures and Tables

**Table 1:** Parentage analysis.

Code		Published Parent Combination ^1^		Suggested Parent Combination ^2^		Comment
	Accession	Mother	Father	Parent 1	Parent 2	
PA067	Cacanska Lepotica (c)	Wangenheim	Pozegaca	Ruth Gerstetter	Stanley (c)	
PA010	Stanley (c)	Agenska	Grand Duke	Agenska	?	
PA335	Anna Spaeth	?	?	Zelena Renkloda	Svestka domaci	
PA370	Aprimira	Mirabelle von Herrenhausen	?	?	?	
PA248	Belgicka modra	?	?	?	?	clone of Svestka domaci
PA371	Bellamira	Cacanska Najbolja	Mirabelka Nancy	Cacanska Najbolja	Mirabelka Nancy	
PA025	Bonne de Bry	?	?	?	?	
PA011	Bystricka muskatova	?	?	?	?	clone of Svestka domaci
PA159	Cacanska rodna	Stanley (c)	Pozegaca	Stanley (c)	Pozegaca	
PA091	Carpatin	Tuleu Gras	Early Rivers	Tuleu Gras	Ruth Gerstetter	
PA082	Dambovita	?	?	Tuleu Gras	Malvazinka	
PA084	Esslingenska svestka	?	?	Viola Szini Diapre	Svestka domaci	
PA181	Excalibur	Althanova Renkloda	?	Althanova Renkloda	Washington	
PA208	Gabrovska	Kjustendilska modra	Montfortska	Montfortska	Svestka domaci	
PA162	Hamanova svestka	?	?	?	?	
PA017	Herman	Ruth Gerstetter	Czar	Ruth Gerstetter	Czar	
PA209	Chodovlicka	?	?	?	?	
PA069	Katalonsky spendlik	?	?	?	?	
PA427	Mirabelka Flotova	?	?	?	?	
PA174	Mirabelka Jaune de Plovdiv	?	?	?	?	
PA128	Mirabelka Metska	?	?	?	?	
PA670	Mirabelka Nancy	?	?	?	?	clone of Mirabelka Metska
PA060	Opal	Oullinska	Early Favourite	Oullinska	?	
PA276	Ortenauer	?	?	?	?	
PA143	Schueleho rana	?	?	?	?	
PA238	Svestka domaci	?	?	?	?	
PA153	Timocanka	Stanley (c)	California Blue	Stanley (c)	California Blue	
PA315	Top 2000	Stanley (c)	?	Stanley (c)	Auerbacher	
PA157	Topgigant	Cacanska Najbolja	President	Cacanska Najbolja	President	
PA281	Topking	Cacanska Najbolja	Italian Prune	Cacanska Najbolja	Auerbacher	
PA144	Topstar	Ersinger	Cacanska Najbolja	President	Cacanska Najbolja	
PA320	Toptaste	Valor	German Prune	Valor	Cacanska Najbolja	
PA294	Tuleu Gras	?	?	?	?	
PA184	Valjevka	d‘Agen 707	Stanley (c)	?	Stanley (c)	
PA012	Valor	Imperial Epineuse	Grand Duke	?	?	
PA210	Vankova Chrudimska	?	?	?	?	

^1.^ Published parent combinations were taken from Butac [20]; ^2^ suggested parent combinations were obtained by parentage analysis in software POLYGENE [21]. ? means unknown.

**Table 2:** External pomological traits.

Cultivars	Fruit Shape	Fruit Color	Flesh Color	Wax Bloom	Bruising Resistance
Blue group ^1^					
Cacanska Lepotica (c)	7.0 ± 0.0	8.0 ± 0.0	3.0 ± 0.0	7.5 ± 0.5	7.0 ± 0.0
Stanley (c)	6.0 ± 0.0	8.0 ± 0.0	4.0 ± 0.0	8.0 ± 0.0	6.0 ± 1.0
Aprimira	6.0 ± 2.3	2.0 ± 0.0	6.8 ± 1.3	2.5 ± 0.9	5.0 ± 1.2
Carpatin	7.0 ± 0.0	8.0 ± 0.0	3.6 ± 0.5	6.8 ± 0.4	7.0 ± 0.0
Dambovita	4.0 ± 0.0	8.0 ± 0.0	4.5 ± 0.5	7.5 ± 0.5	6.5 ± 0.9
Herman	3.8 ± 1.6	7.6 ± 0.5	4.2 ± 1.0	6.6 ± 0.5	5.4 ± 0.5
Katalonsky spendlik	2.0 ± 0.0	2.0 ± 0.0	5.5 ± 0.5	2.5 ± 0.5	5.3 ± 1.3
Timocanka	2.0 ± 0.0	6.4 ± 0.8	5.6 ± 0.8	7.0 ± 0.0	6.2 ± 0.4
Topgigant	7.0 ± 0.0	8.0 ± 0.0	3.3 ± 0.4	6.8 ± 0.4	5.5 ± 0.9
Topstar	4.0 ± 0.0	7.6 ± 0.8	5.8 ± 0.4	6.8 ± 0.4	6.8 ± 0.4
Toptaste	6.8 ± 0.4	8.0 ± 0.0	6.2 ± 0.4	7.4 ± 0.5	7.0 ± 0.0
Valor	4.0 ± 0.0	8.0 ± 0.0	6.0 ± 1.5	7.8 ± 0.4	5.8 ± 0.7
Red group ^2^					
Anna Spaeth	2.0 ± 0.0	6.0 ± 1.0	5.3 ± 0.8	6.5 ± 0.5	5.8 ± 0.4
Belgicka modra	5.0 ± 0.0	8.0 ± 0.0	3.5 ± 0.5	8.0 ± 0.0	6.0 ± 1.0
Bystricka muskatova	5.0 ± 0.0	7.6 ± 0.8	5.4 ± 0.5	7.8 ± 0.4	7.0 ± 0.0
Cacanska rodna	5.0 ± 0.0	7.3 ± 1.3	3.0 ± 0.0	7.5 ± 0.9	7.0 ± 0.0
Esslingenska svestka	6.0 ± 0.0	7.4 ± 0.8	5.2 ± 0.4	7.0 ± 0.0	6.0 ± 0.6
Excalibur	2.0 ± 0.0	3.4 ± 0.8	6.4 ± 0.5	3.6 ± 0.5	6.0 ± 0.0
Gabrovska	5.0 ± 0.0	7.4 ± 0.8	6.0 ± 0.0	7.2 ± 0.4	6.0 ± 1.7
Hamanova svestka	5.0 ± 0.0	8.0 ± 0.0	4.0 ± 0.0	5.8 ± 0.4	8.0 ± 0.0
Chodovlicka	3.8 ± 1.0	7.6 ± 0.5	5.0 ± 0.9	6.2 ± 0.7	6.4 ± 0.5
Ortenauer	5.0 ± 0.0	8.0 ± 0.0	5.2 ± 0.4	7.8 ± 0.4	6.6 ± 0.8
Schueleho rana	5.3 ± 0.4	7.5 ± 0.9	3.8 ± 1.3	6.5 ± 0.9	6.5 ± 0.5
Svestka domaci	4.3 ± 1.3	8.0 ± 0.0	3.8 ± 0.4	7.8 ± 0.4	6.5 ± 0.5
Top 2000	5.0 ± 0.0	7.6 ± 0.8	5.0 ± 0.0	7.8 ± 0.4	6.4 ± 0.5
Topking	4.0 ± 0.0	7.6 ± 0.8	5.4 ± 0.8	7.6 ± 0.5	6.4 ± 1.2
Tuleu Gras	6.0 ± 0.0	8.0 ± 0.0	6.0 ± 0.0	7.5 ± 0.5	6.8 ± 0.4
Valjevka	7.2 ± 1.0	6.4 ± 0.8	5.2 ± 0.4	7.8 ± 0.4	7.4 ± 0.5
Vankova Chrudimska	4.0 ± 0.0	6.6 ± 0.5	4.2 ± 0.4	6.6 ± 0.5	7.0 ± 0.0
Green group ^3^					
Bellamira	7.0 ± 0.0	2.7 ± 0.5	6.3 ± 0.9	5.7 ± 0.5	6.3 ± 0.5
Bonne de Bry	1.0 ± 0.0	7.0 ± 0.0	3.0 ± 0.0	6.4 ± 0.5	5.8 ± 0.7
Mirabelka Flotova	1.0 ± 0.0	2.0 ± 0.0	6.8 ± 0.4	2.8 ± 0.4	2.8 ± 2.2
Mirabelka Jaune de Plovdiv	2.0 ± 0.0	2.0 ± 0.0	5.8 ± 0.4	2.4 ± 0.5	4.4 ± 0.5
Opal	2.0 ± 0.0	6.2 ± 0.7	7.0 ± 0.6	7.0 ± 0.0	5.0 ± 0.0
Orange group ^4^					
Mirabelka Nancy	1.0 ± 0.0	3.0 ± 0.0	5.0 ± 0.0	7.0 ± 0.0	6.0 ± 0.0
Mirabelka Metska	2.0 ± 1.0	3.8 ± 1.9	5.0 ± 0.0	5.0 ± 1.4	7.0 ± 0.0

Data are presented as mean ± SD; ^1^—modern European and North American varieties; ^2^—traditional landraces and several Balkan and Romanian cultivars; ^3^—gages and mirabelles; ^4^—subgroup contains two mirabelles; the groups were created based on genetic analyses.

**Table 3:** Internal structural pomological characteristics.

Cultivars	Skin Firmness	Skin Separability	Flesh Firmness	Flesh Texture	Stone Separability
Blue group ^1^					
Cacanska Lepotica (c)	7.0 ± 0.0	4.8 ± 0.4	7.0 ± 0.0	5.8 ± 0.4	8.5 ± 0.5
Stanley (c)	6.3 ± 0.4	5.0 ± 1.0	5.5 ± 0.9	5.5 ± 0.5	6.8 ± 0.8
Aprimira	4.3 ± 0.4	4.5 ± 0.9	5.3 ± 1.1	6.0 ± 1.0	7.8 ± 0.8
Carpatin	6.2 ± 0.7	6.4 ± 0.5	5.6 ± 0.5	7.0 ± 0.0	8.2 ± 1.6
Dambovita	7.3 ± 1.1	5.8 ± 1.9	7.0 ± 0.0	5.8 ± 0.4	6.3 ± 0.4
Herman	4.6 ± 0.8	7.2 ± 0.4	5.6 ± 0.8	6.8 ± 0.4	7.6 ± 0.5
Katalonsky spendlik	5.8 ± 0.4	5.3 ± 0.4	6.3 ± 0.8	5.3 ± 0.4	8.3 ± 0.4
Timocanka	6.6 ± 1.2	6.6 ± 1.2	5.2 ± 1.3	5.2 ± 0.4	5.8 ± 2.4
Topgigant	7.0 ± 0.0	6.0 ± 0.0	5.8 ± 0.4	6.0 ± 0.0	8.0 ± 0.0
Topstar	6.0 ± 0.0	4.8 ± 0.4	5.8 ± 0.4	6.2 ± 0.4	9.0 ± 0.0
Toptaste	6.6 ± 0.8	5.0 ± 0.0	7.0 ± 0.0	6.6 ± 0.5	7.6 ± 0.8
Valor	5.0 ± 0.0	5.0 ± 0.9	6.4 ± 0.8	6.4 ± 0.5	7.2 ± 0.4
Red group ^2^					
Anna Spaeth	5.3 ± 0.4	5.8 ± 0.4	5.5 ± 0.5	7.0 ± 0.0	5.8 ± 1.1
Belgicka modra	7.0 ± 0.0	3.0 ± 1.0	7.0 ± 1.0	4.5 ± 1.5	8.0 ± 1.0
Bystricka muskatova	5.8 ± 0.4	5.0 ± 2.0	6.6 ± 0.8	5.8 ± 0.4	9.0 ± 0.0
Cacanska rodna	6.8 ± 0.4	3.3 ± 0.4	7.0 ± 0.7	5.3 ± 1.1	8.8 ± 0.4
Esslingenska svestka	5.0 ± 0.0	5.0 ± 0.0	4.4 ± 0.5	6.6 ± 0.5	6.8 ± 1.0
Excalibur	6.0 ± 0.0	4.4 ± 0.5	6.2 ± 1.0	5.0 ± 0.9	8.6 ± 0.5
Gabrovska	7.0 ± 0.9	4.2 ± 0.7	7.4 ± 0.5	4.8 ± 1.0	9.0 ± 0.0
Hamanova svestka	6.0 ± 0.6	3.4 ± 0.8	7.0 ± 0.0	7.0 ± 0.0	9.0 ± 0.0
Chodovlicka	4.8 ± 0.7	5.6 ± 0.5	5.6 ± 1.4	6.4 ± 0.5	6.2 ± 0.4
Ortenauer	6.6 ± 0.5	4.2 ± 2.4	7.0 ± 1.1	4.8 ± 0.7	7.6 ± 0.8
Schueleho rana	6.0 ± 0.0	5.0 ± 0.0	6.5 ± 0.5	5.5 ± 0.9	8.5 ± 0.5
Svestka domaci	5.8 ± 0.4	4.8 ± 0.8	6.8 ± 0.4	6.0 ± 0.0	8.0 ± 0.0
Top 2000	6.4 ± 0.5	4.4 ± 0.5	5.6 ± 1.2	6.2 ± 0.4	8.8 ± 0.4
Topking	6.0 ± 1.5	4.2 ± 0.4	6.2 ± 0.7	6.2 ± 0.4	7.6 ± 0.5
Tuleu Gras	7.0 ± 0.0	4.8 ± 0.4	5.8 ± 0.4	5.0 ± 0.0	7.3 ± 1.3
Valjevka	6.4 ± 0.5	5.0 ± 0.0	6.6 ± 0.5	5.6 ± 0.5	8.2 ± 0.4
Vankova Chrudimska	6.0 ± 0.0	4.4 ± 0.5	4.6 ± 1.2	6.2 ± 0.7	7.6 ± 1.2
Green group ^3^					
Bellamira	7.3 ± 0.5	4.3 ± 0.9	7.3 ± 0.5	4.0 ± 0.0	9.0 ± 0.0
Bonne de Bry	5.0 ± 0.0	5.0 ± 0.0	5.4 ± 1.0	6.4 ± 0.5	7.4 ± 1.2
Mirabelka Flotova	5.2 ± 0.4	5.4 ± 0.5	4.0 ± 0.0	6.4 ± 0.5	5.0 ± 0.6
Mirabelka Jaune de Plovdiv	5.8 ± 0.4	4.4 ± 0.5	5.0 ± 0.0	5.8 ± 0.4	7.4 ± 0.5
Opal	5.2 ± 0.7	7.0 ± 0.6	5.2 ± 0.7	6.8 ± 0.4	9.0 ± 0.0
Orange group ^4^					
Mirabelka Nancy	5.7 ± 0.5	6.0 ± 0.0	7.0 ± 0.0	5.0 ± 0.0	8.3 ± 0.9
Mirabelka Metska	6.0 ± 0.0	4.8 ± 2.2	6.0 ± 0.7	6.8 ± 0.4	8.8 ± 0.4

Data are presented as mean ± SD; ^1^—modern European and North American varieties; ^2^—traditional landraces and several Balkan and Romanian cultivars; ^3^—gages and mirabelles; ^4^—subgroup contains two mirabelles; the groups were created based on genetic analyses.

**Table 4:** Internal organoleptic pomological characteristics.

Cultivars	Orthonasal Aroma of Flesh	Taste of Skin	Juiciness	Taste of Fruit	Retronasal Aroma of Fruit	Perceived Acidity/Sweetness Balance
Blue group ^1^						
Cacanska Lepotica (c)	4.0 ± 0.0	5.0 ± 0.0	6.0 ± 0.0	6.0 ± 1.0	6.0 ± 0.0	6.3 ± 0.4
Stanley (c)	4.3 ± 0.4	5.5 ± 0.5	5.8 ± 0.4	6.5 ± 0.5	6.0 ± 0.0	7.3 ± 0.8
Aprimira	6.0 ± 0.0	5.5 ± 0.9	5.8 ± 0.8	6.8 ± 0.4	6.5 ± 0.5	7.8 ± 0.4
Carpatin	5.4 ± 0.8	6.0 ± 0.0	7.0 ± 0.0	7.0 ± 0.0	6.8 ± 0.4	7.4 ± 0.5
Dambovita	5.8 ± 0.4	5.0 ± 0.0	7.3 ± 0.4	6.5 ± 0.9	7.0 ± 0.0	5.8 ± 1.6
Herman	4.8 ± 0.4	5.8 ± 0.4	6.2 ± 0.7	6.0 ± 0.0	5.8 ± 0.4	6.4 ± 0.5
Katalonsky spendlik	5.3 ± 1.1	5.3 ± 0.4	6.8 ± 0.4	7.3 ± 0.4	6.8 ± 0.4	7.0 ± 0.0
Timocanka	5.6 ± 0.5	6.6 ± 0.8	7.0 ± 0.0	6.4 ± 0.5	6.2 ± 0.7	7.6 ± 0.5
Topgigant	5.3 ± 0.4	4.0 ± 0.0	5.8 ± 0.4	6.0 ± 0.0	5.0 ± 0.0	6.0 ± 0.0
Topstar	5.8 ± 0.4	5.0 ± 0.0	4.0 ± 0.0	6.2 ± 0.4	5.8 ± 0.4	7.0 ± 0.0
Toptaste	4.0 ± 0.0	5.4 ± 0.5	6.8 ± 0.7	7.6 ± 0.8	7.0 ± 0.0	7.6 ± 0.5
Valor	5.2 ± 0.4	4.6 ± 0.5	7.0 ± 0.0	7.0 ± 0.0	6.4 ± 0.5	7.6 ± 0.5
Red group ^2^						
Anna Spaeth	4.8 ± 0.4	5.3 ± 0.4	7.0 ± 0.0	7.0 ± 0.0	7.0 ± 0.0	7.8 ± 0.4
Belgicka modra	6.0 ± 0.0	5.0 ± 1.0	5.5 ± 0.5	5.8 ± 0.8	5.8 ± 0.8	6.3 ± 0.4
Bystricka muskatova	5.4 ± 0.5	5.0 ± 0.0	4.4 ± 1.7	6.2 ± 0.7	6.2 ± 0.7	6.6 ± 0.8
Cacanska rodna	4.3 ± 1.3	5.0 ± 0.0	6.8 ± 0.4	5.8 ± 0.8	6.0 ± 0.0	5.5 ± 0.5
Chodovlicka	6.2 ± 0.4	5.0 ± 0.0	6.6 ± 0.5	6.2 ± 0.4	5.8 ± 1.0	6.6 ± 0.5
Esslingenska svestka	5.4 ± 0.5	4.8 ± 1.0	7.0 ± 0.0	7.0 ± 0.6	7.2 ± 1.0	6.6 ± 0.5
Excalibur	5.4 ± 0.5	4.8 ± 0.7	6.4 ± 0.5	7.2 ± 0.4	6.4 ± 0.5	7.8 ± 0.4
Gabrovska	5.8 ± 0.4	4.8 ± 1.0	5.8 ± 0.4	7.0 ± 0.0	6.2 ± 0.7	7.0 ± 0.0
Hamanova svestka	6.0 ± 0.0	6.0 ± 0.0	6.8 ± 1.0	7.8 ± 0.4	7.2 ± 0.4	7.2 ± 0.4
Ortenauer	5.6 ± 0.5	4.2 ± 1.6	6.0 ± 0.6	6.0 ± 0.6	5.8 ± 0.4	6.0 ± 0.6
Schueleho rana	4.3 ± 0.4	4.8 ± 0.4	5.8 ± 1.1	5.3 ± 0.8	4.8 ± 0.4	5.3 ± 0.8
Svestka domaci	4.8 ± 0.8	5.0 ± 1.7	6.3 ± 0.4	6.3 ± 0.4	5.8 ± 0.4	5.8 ± 0.4
Top 2000	4.6 ± 0.5	5.4 ± 0.5	6.0 ± 0.0	6.6 ± 0.5	6.0 ± 0.0	6.6 ± 0.5
Topking	5.4 ± 0.5	3.4 ± 0.5	6.2 ± 0.7	6.6 ± 0.5	6.6 ± 0.5	6.6 ± 0.5
Tuleu Gras	5.3 ± 0.4	5.0 ± 0.0	7.0 ± 0.0	7.0 ± 0.0	6.8 ± 0.4	6.8 ± 0.4
Valjevka	5.2 ± 0.4	4.6 ± 0.5	6.0 ± 0.0	6.6 ± 0.5	6.6 ± 0.5	6.4 ± 0.5
Vankova Chrudimska	5.0 ± 0.9	5.8 ± 0.4	6.6 ± 0.5	6.6 ± 0.5	6.6 ± 0.5	7.8 ± 0.4
Green group ^3^						
Bellamira	5.7 ± 0.5	5.0 ± 0.0	6.0 ± 0.0	7.7 ± 0.5	7.0 ± 0.0	7.7 ± 0.5
Bonne de Bry	5.0 ± 0.0	4.6 ± 0.5	6.4 ± 0.5	6.0 ± 0.6	6.2 ± 0.7	6.0 ± 0.9
Mirabelka Flotova	4.8 ± 0.4	6.0 ± 0.0	6.0 ± 0.6	6.4 ± 0.5	6.4 ± 0.5	6.6 ± 0.5
Mirabelka Jaune de Plovdiv	4.6 ± 0.5	5.0 ± 0.0	6.0 ± 0.0	6.4 ± 0.5	5.8 ± 0.4	6.0 ± 0.0
Opal	5.4 ± 0.5	6.6 ± 0.8	7.0 ± 0.0	7.2 ± 0.4	6.6 ± 0.5	7.4 ± 0.5
Orange group ^4^						
Mirabelka Nancy	5.0 ± 0.0	6.0 ± 0.0	6.0 ± 0.0	5.0 ± 0.0	5.0 ± 0.0	6.3 ± 0.5
Mirabelka Metska	5.0 ± 0.0	6.5 ± 0.9	5.5 ± 2.6	5.5 ± 1.5	5.3 ± 1.3	7.3 ± 1.3

Data are presented as mean ± SD; ^1^—modern European and North American varieties; ^2^—traditional landraces and several Balkan and Romanian cultivars; ^3^—gages and mirabelles; ^4^—subgroup contains two mirabelles; the groups were created based on genetic analyses.

**Table 5:** Measurable pomological traits.

Cultivars	Fruit Weight	Fruit Height	Fruit Width	Fruit Thickness	Pedicel Length	Soluble Solids Content
Blue group ^1^						
Cacanska Lepotica (c)	39.0 ± 4.6	43.2 ± 3.0	38.6 ± 1.5	37.2 ± 1.8	17.8 ± 2.1	14.5 ± 1.0
Stanley (c)	37.1 ± 2.9	50.5 ± 1.7	37.6 ± 0.8	35.5 ± 1.1	20.0 ± 2.6	16.6 ± 2.5
Aprimira	32.2 ± 7.8	41.2 ± 5.1	36.6 ± 4.2	32.8 ± 4.9	17.3 ± 6.2	22.6 ± 0.5
Carpatin	48.0 ± 6.2	47.7 ± 5.4	36.2 ± 5.5	37.0 ± 5.7	14.5 ± 2.5	15.0 ± 1.9
Dambovita	43.3 ± 4.4	49.0 ± 3.1	42.5 ± 4.9	40.9 ± 4.1	24.9 ± 12.7	17.3 ± 4.3
Herman	32.2 ± 7.7	42.4 ± 2.4	39.0 ± 1.9	34.3 ± 6.2	10.6 ± 1.6	12.0 ± 3.2
Katalonsky spendlik	31.4 ± 3.4	38.2 ± 1.4	35.1 ± 1.5	33.7 ± 1.1	12.0 ± 0.8	15.2 ± 1.7
Timocanka	71.0 ± 8.4	51.9 ± 2.2	48.2 ± 2.2	46.2 ± 3.1	15.7 ± 3.6	15.4 ± 2.2
Topgigant	78.7 ± 7.9	58.2 ± 3.9	46.1 ± 3.8	44.8 ± 3.4	18.7 ± 1.4	16.8 ± 1.4
Topstar	48.8 ± 3.0	49.6 ± 3.2	38.9 ± 4.2	40.3 ± 5.5	21.0 ± 5.3	15.1 ± 3.2
Toptaste	46.7 ± 9.2	49.5 ± 2.2	39.5 ± 2.5	38.2 ± 2.1	19.8 ± 1.8	22.3 ± 4.2
Valor	51.4 ± 7.4	50.9 ± 2.1	42.8 ± 2.9	40.7 ± 2.4	20.4 ± 2.2	19.7 ± 1.9
Red group ^2^						
Anna Spaeth	45.7 ± 3.9	46.0 ± 0.3	39.5 ± 0.2	38.8 ± 3.0	16.5 ± 0.4	23.9 ± 3.2
Belgicka modra	16.4 ± 1.7	36.6 ± 1.9	27.9 ± 2.3	32.2 ± 8.3	16.6 ± 1.3	19.6 ± 1.9
Bystricka muskatova	18.5 ± 3.9	41.0 ± 3.0	29.1 ± 2.4	28.3 ± 2.0	17.7 ± 2.0	19.1 ± 1.4
Cacanska rodna	34.9 ± 2.8	46.1 ± 2.4	36.3 ± 1.4	34.1 ± 2.6	23.3 ± 3.6	17.0 ± 1.2
Esslingenska svestka	19.6 ± 3.3	37.1 ± 2.6	29.2 ± 1.6	28.9 ± 1.7	19.4 ± 4.2	18.7 ± 3.0
Excalibur	68.4 ± 14.4	51.4 ± 3.1	48.8 ± 3.4	48.1 ± 3.1	26.6 ± 2.6	16.0 ± 3.1
Gabrovska	33.5 ± 4.1	49.6 ± 2.2	37.4 ± 5.5	36.1 ± 3.8	18.8 ± 2.1	16.3 ± 2.6
Hamanova svestka	21.7 ± 1.6	40.5 ± 1.8	31.1 ± 1.1	30.5 ± 1.3	17.7 ± 0.9	18.8 ± 3.2
Chodovlicka	30.4 ± 5.2	44.1 ± 1.5	35.2 ± 4.1	34.4 ± 2.5	21.0 ± 1.9	17.9 ± 2.3
Ortenauer	28.7 ± 1.8	28.7 ± 1.8	48.9 ± 3.4	35.8 ± 6.0	17.3 ± 2.8	17.6 ± 1.3
Schueleho rana	24.4 ± 2.6	44.3 ± 2.3	30.1 ± 1.4	29.4 ± 3.2	21.6 ± 1.0	17.7 ± 2.3
Svestka domaci	18.9 ± 2.6	41.7 ± 3.6	27.6 ± 3.0	28.3 ± 1.5	18.8 ± 2.0	16.4 ± 0.2
Top 2000	26.4 ± 5.0	42.1 ± 2.0	31.9 ± 2.3	31.2 ± 2.8	19.6 ± 0.8	21.2 ± 3.2
Topking	31.2 ± 3.8	43.6 ± 6.2	30.9 ± 6.7	30.0 ± 7.9	18.9 ± 1.0	24.8 ± 3.6
Tuleu Gras	31.6 ± 2.7	44.3 ± 1.0	33.5 ± 0.6	33.1 ± 2.8	14.8 ± 1.3	20.0 ± 1.5
Valjevka	34.4 ± 6.8	51.5 ± 2.1	36.6 ± 1.7	35.8 ± 3.2	18.2 ± 1.3	17.6 ± 2.8
Vankova Chrudimska	35.3 ± 3.5	46.8 ± 2.1	36.6 ± 1.1	36.0 ± 1.7	17.7 ± 1.5	21.9 ± 3.8
Green group ^3^						
Bellamira	22.1 ± 2.4	32.2 ± 1.3	29.2 ± 0.7	29.1 ± 2.6	19.5 ± 2.0	21.4 ± 5.0
Bonne de Bry	18.2 ± 4.8	27.9 ± 2.2	29.9 ± 3.6	30.4 ± 3.4	17.7 ± 1.2	16.2 ± 2.0
Mirabelka Flotova	15.6 ± 2.6	25.5 ± 5.6	25.4 ± 5.7	25.1 ± 5.6	14.5 ± 1.6	16.5 ± 1.5
Mirabelka Jaune de Plovdiv	14.2 ± 3.1	30.3 ± 3.8	28.0 ± 2.9	25.9 ± 2.5	19.7 ± 1.8	20.9 ± 3.7
Opal	33.5 ± 4.1	39.6 ± 1.0	36.9 ± 1.6	36.5 ± 1.5	17.2 ± 2.2	15.8 ± 1.6
Orange group ^4^						
Mirabelka Nancy	12.9 ± 0.6	27.8 ± 0.3	26.6 ± 0.7	25.8 ± 0.6	17.6 ± 1.3	21.8 ± 1.4
Mirabelka Metska	9.3 ± 2.2	26.6 ± 3.4	24.5 ± 2.1	24.0 ± 2.3	14.6 ± 4.2	18.0 ± 3.5

Data are presented as mean ± SD; ^1^—modern European and North American varieties; ^2^—traditional landraces and several Balkan and Romanian cultivars; ^3^—gages and mirabelles; ^4^—subgroup contains two mirabelles; the groups were created based on genetic analyses.

**Table 6:** Correlation dependence.

	Fruit Color	Flesh Color	Wax Bloom	Bruising Resistance	Skin Firmness	Skin Separability	Flesh Firmness	Flesh Texture	Stone Separability	Orthonasal Aroma of Flesh	Taste of Skin	Juiciness	Taste of Fruit	Retronasal Aroma of Fruit	Perceived Acidity/Sweetness Balance	Fruit Weight	Fruit Height	Fruit Width	Fruit Thickness	Pedicel Length	Soluble Solids Content
Fruit shape	N.S.	N.S.	N.S.	N.S.	N.S.	N.S.	N.S.	N.S.	N.S.	N.S.	N.S.	N.S.	N.S.	N.S.	N.S.	N.S.	N.S.	N.S.	N.S.	N.S.	N.S.
Fruit color	-	N.S.	0.9	N.S.	N.S.	N.S.	N.S.	N.S.	N.S.	N.S.	N.S.	N.S.	N.S.	N.S.	N.S.	N.S.	N.S.	N.S.	N.S.	N.S.	N.S.
Flesh color	-	-	N.S.	N.S.	N.S.	N.S.	N.S.	N.S.	N.S.	N.S.	N.S.	N.S.	N.S.	N.S.	N.S.	N.S.	N.S.	N.S.	N.S.	N.S.	N.S.
Wax bloom	-	-	-	N.S.	N.S.	N.S.	N.S.	N.S.	N.S.	N.S.	N.S.	N.S.	N.S.	N.S.	N.S.	N.S.	N.S.	N.S.	N.S.	N.S.	N.S.
Bruising resistance	-	-	-	-	N.S.	N.S.	N.S.	N.S.	N.S.	N.S.	N.S.	N.S.	N.S.	N.S.	N.S.	N.S.	N.S.	N.S.	N.S.	N.S.	N.S.
Skin firmness	-	-	-	-	-	N.S.	N.S.	N.S.	N.S.	N.S.	N.S.	N.S.	N.S.	N.S.	N.S.	N.S.	N.S.	N.S.	N.S.	N.S.	N.S.
Skin separability	-	-	-	-	-	-	N.S.	N.S.	N.S.	N.S.	N.S.	N.S.	N.S.	N.S.	N.S.	N.S.	N.S.	N.S.	N.S.	N.S.	N.S.
Flesh firmness	-	-	-	-	-	-	-	N.S.	N.S.	N.S.	N.S.	N.S.	N.S.	N.S.	N.S.	N.S.	N.S.	N.S.	N.S.	N.S.	N.S.
Flesh texture	-	-	-	-	-	-	-	-	N.S.	N.S.	N.S.	N.S.	N.S.	N.S.	N.S.	N.S.	N.S.	N.S.	N.S.	N.S.	N.S.
Stone separability	-	-	-	-	-	-	-	-	-	N.S.	N.S.	N.S.	N.S.	N.S.	N.S.	N.S.	N.S.	N.S.	N.S.	N.S.	N.S.
Orthonasal aroma of flesh	-	-	-	-	-	-	-	-	-	-	N.S.	N.S.	N.S.	N.S.	N.S.	N.S.	N.S.	N.S.	N.S.	N.S.	N.S.
Taste of skin	-	-	-	-	-	-	-	-	-	-	-	N.S.	N.S.	N.S.	N.S.	N.S.	N.S.	N.S.	N.S.	N.S.	N.S.
Juiciness	-	-	-	-	-	-	-	-	-	-	-	-	N.S.	N.S.	N.S.	N.S.	N.S.	N.S.	N.S.	N.S.	N.S.
Taste of fruit	-	-	-	-	-	-	-	-	-	-	-	-	-	0.8	0.7	N.S.	N.S.	N.S.	N.S.	N.S.	N.S.
Retronasal aroma of fruit	-	-	-	-	-	-	-	-	-	-	-	-	-	-	N.S.	N.S.	N.S.	N.S.	N.S.	N.S.	N.S.
Perceived acidity/sweetness balance	-	-	-	-	-	-	-	-	-	-	-	-	-	-	-	N.S.	N.S.	N.S.	N.S.	N.S.	N.S.
Fruit weight	-	-	-	-	-	-	-	-	-	-	-	-	-	-	-	-	0.8	0.9	0.9	N.S.	N.S.
Fruit height	-	-	-	-	-	-	-	-	-	-	-	-	-	-	-	-	-	0.8	0.8	N.S.	N.S.
Fruit width	-	-	-	-	-	-	-	-	-	-	-	-	-	-	-	-	-	-	1.0	N.S.	N.S.
Fruit thickness	-	-	-	-	-	-	-	-	-	-	-	-	-	-	-	-	-	-	-	N.S.	N.S.
Pedicel length	-	-	-	-	-	-	-	-	-	-	-	-	-	-	-	-	-	-	-	-	N.S.

Only coefficients greater than or equal to 0.7 are shown; the others are presented as non-significant correlations (N.S.).

**Table 7:** Result of permutation-based ANOVA; only statistically significant results are shown.

Trait	*p*-Value
Fruit shape	0.0071
Fruit color	0.0031
Flesh color	N.S.
Wax bloom	N.S.
Bruising resistance	0.0058
Skin firmness	N.S.
Skin separability	0.02
Flesh firmness	N.S.
Flesh texture	N.S.
Stone separability	N.S.
Orthonasal aroma of flesh	N.S.
Taste of skin	0.047
Juiciness	N.S.
Taste of fruit	0.033
Retronasal aroma of fruit	N.S.
Perceived acidity/sweetness balance	N.S.
Fruit weight (g)	0.0008
Fruit height (mm)	0.0000001
Fruit width (mm)	0.0000001
Fruit thickness (mm)	0.0003
Pedicel length (mm)	N.S.
Soluble solids content (°Brix)	N.S.

Only *p*-values lower than or equal to 0.05 are shown (rounded to two significant digits); the others are presented as non-significant (N.S.).

**Table 8:** Statistical evaluation of inter-group differences by pairwise permutation test.

	Blue (n = 12) × Red (n = 17)		Blue (n = 12) × Green (n = 5)		Blue (n = 12) × Orange (n = 2)		Red (n = 17) × Green (n = 5)		Red (n = 17) × Orange (n = 2)		Green (n = 5) × Orange (n = 2)	
Trait	Mean × Mean	*p*-Value	Mean × Mean	*p*-Value	Mean × Mean	*p*-Value	Mean × Mean	*p*-Value	Mean × Mean	*p*-Value	Mean × Mean	*p*-Value
Fruit shape	4.97 × 4.68	N.S.	4.97 × 2.60	N.S.	4.97 × 1.50	0.035	4.68 × 2.60	0.022	4.68 × 1.50	0.018	2.60 × 1.50	N.S.
Fruit color	6.80 × 7.20	N.S.	6.80 × 3.97	0.02	6.80 × 3.38	N.S.	7.20 × 3.97	0.0008	7.20 × 3.38	0.011	3.97 × 3.38	N.S.
Flesh color	4.87 × 4.84	N.S.	4.87 × 5.79	N.S.	4.87 × 5.00	N.S.	4.84 × 5.79	N.S.	4.84 × 5.00	N.S.	5.79 × 5.00	N.S.
Wax bloom	6.43 × 7.00	N.S.	6.43 × 4.85	N.S.	6.43 × 6.00	N.S.	7.00 × 4.85	0.0066	7.00 × 6.00	N.S.	4.85 × 6.00	N.S.
Bruising resistance	6.12 × 6.57	N.S.	6.12 × 4.87	0.023	6.12 × 6.50	N.S.	6.57 × 4.87	0.0008	6.57 × 6.50	N.S.	4.87 × 6.50	N.S.
Skin firmness	6.04 × 6.10	N.S.	6.04 × 5.71	N.S.	6.04 × 5.83	N.S.	6.10 × 5.71	N.S.	6.10 × 5.83	N.S.	5.71 × 5.83	N.S.
Skin separability	5.52 × 4.49	0.0014	5.52 × 5.23	N.S.	5.52 × 5.38	N.S.	4.49 × 5.23	N.S.	4.49 × 5.38	N.S.	5.23 × 5.38	N.S.
Flesh firmness	6.03 × 6.22	N.S.	6.03 × 5.39	N.S.	6.03 × 6.50	N.S.	6.22 × 5.39	N.S.	6.22 × 6.50	N.S.	5.39 × 6.50	N.S.
Flesh texture	6.04 × 5.76	N.S.	6.04 × 5.88	N.S.	6.04 × 5.88	N.S.	5.76 × 5.88	N.S.	5.76 × 5.88	N.S.	5.88 × 5.88	N.S.
Stone separability	7.58 × 7.92	N.S.	7.58 × 7.56	N.S.	7.58 × 8.54	N.S.	7.92 × 7.56	N.S.	7.92 × 8.54	N.S.	7.56 × 8.54	N.S.
Orthonasal aroma of flesh	5.11 × 5.25	N.S.	5.11 × 5.09	N.S.	5.11 × 5.00	N.S.	5.25 × 5.09	N.S.	5.25 × 5.00	N.S.	5.09 × 5.00	N.S.
Taste of skin	5.30 × 4.93	N.S.	5.30 × 5.44	N.S.	5.30 × 6.25	N.S.	4.93 × 5.44	N.S.	4.93 × 6.25	0.02	5.44 × 6.25	N.S.
Juiciness	6.27 × 6.24	N.S.	6.27 × 6.28	N.S.	6.27 × 5.75	N.S.	6.24 × 6.28	N.S.	6.24 × 5.75	N.S.	6.28 × 5.75	N.S.
Taste of fruit	6.60 × 6.52	N.S.	6.60 × 6.73	N.S.	6.60 × 5.25	0.013	6.52 × 6.73	N.S.	6.52 × 5.75	0.011	6.73 × 5.25	N.S.
Retronasal aroma of fruit	6.27 × 6.27	N.S.	6.27 × 6.40	N.S.	6.27 × 5.13	0.034	6.27 × 6.40	N.S.	6.27 × 5.13	0.023	6.40 × 5.13	0.042
Perceived acidity/sweetness balance	6.97 × 6.61	N.S.	6.97 × 6.73	N.S.	6.97 × 6.79	N.S.	6.61 × 6.73	N.S.	6.61 × 6.79	N.S.	6.73 × 6.79	N.S.
Fruit weight (g)	46.64 × 30.58	0.0033	46.64 × 20.70	0.0021	46.64 × 11.11	0.024	30.58 × 20.70	N.S.	30.58 × 11.11	N.S.	20.70 × 11.11	N.S.
Fruit height (mm)	47.69 × 44.44	N.S.	47.69 × 31.12	0.0005	47.69 × 27.17	0.0099	44.44 × 31.12	0.0003	44.44 × 27.17	0.0073	31.12 × 27.17	N.S.
Fruit width (mm)	40.10 × 33.98	0.002	40.10 × 29.88	0.0006	40.10 × 25.23	0.0094	33.98 × 29.88	N.S.	33.98 × 25.53	N.S.	29.88 × 25.33	N.S.
Fruit thickness (mm)	38.48 × 33.47	0.0069	38.48 × 29.40	0.0008	38.48 × 24.92	0.0122	33.47 × 29.40	N.S.	33.47 × 24.92	N.S.	29.40 × 24.92	N.S.
Pedicel length (mm)	17.73 × 19.09	N.S.	17.73 × 17.70	N.S.	17.73 × 16.43	N.S.	19.09 × 17.70	N.S.	19.09 × 16.43	N.S.	17.70 × 16.43	N.S.
Soluble solids content (°Brix)	16.88 × 19.08	N.S.	16.88 × 18.16	N.S.	16.88 × 19.90	N.S.	19.08 × 18.16	N.S.	19.08 × 19.90	N.S.	18.16 × 19.90	N.S.

Only *p*-values lower than or equal to 0.05 are shown (rounded to two significant digits); the others are presented as non-significant (N.S.).

**Table 9:** Statistical evaluation of inter-branch differences by pairwise permutation tests.

	Upper (n = 6) × Middle (n = 14)		Upper (n = 6) × Lower (n = 16)		Middle (n = 14) × Lower (n = 16)	
Trait	Mean × Mean	*p*-Value	Mean × Mean	*p*-Value	Mean × Mean	*p*-Value
Fruit shape	2.33 × 5.34	0.0058	2.33 × 4.15	0.049	5.34 × 4.15	N.S.
Fruit color	2.46 × 7.29	0.0003	2.46 × 7.11	0	7.29 × 7.11	N.S.
Flesh color	5.81 × 4.74	N.S.	5.81 × 4.90	N.S.	4.47 × 4.90	N.S.
Wax bloom	3.70 × 7.23	0.0004	3.70 × 6.81	0.0047	7.23 × 6.81	N.S.
Bruising resistance	5.08 × 6.73	0.0095	5.08 × 6.11	N.S.	6.73 × 6.11	0.0057
Skin firmness	5.44 × 6.47	0.0011	5.44 × 5.82	N.S.	6.47 × 5.82	N.S.
Skin separability	5.05 × 4.33	N.S.	5.05 × 5.53	N.S.	4.33 × 5.53	0.0003
Flesh firmness	5.58 × 6.71	0.02	5.58 × 5.66	N.S.	6.71 × 5.66	0.0001
Flesh texture	5.87 × 5.46	N.S.	5.87 × 6.24	N.S.	5.46 × 6.24	0.0049
Stone separability	7.58 × 8.39	N.S.	7.58 × 7.32	N.S.	8.39 × 7.35	0.0035
Orthonasal aroma of flesh	5.11 × 5.24	N.S.	5.11 × 5.13	N.S.	5.24 × 5.13	N.S.
Taste of skin	5.71 × 4.84	0.0018	5.71 × 5.32	N.S.	4.84 × 5.32	N.S.
Juiciness	6.00 × 5.89	N.S.	6.00 × 6.61	0.015	5.89 × 6.61	0.0089
Taste of fruit	6.22 × 6.43	N.S.	6.22 × 6.68	N.S.	6.43 × 6.68	N.S.
Retronasal aroma of fruit	5.95 × 6.17	N.S.	5.95 × 6.38	N.S.	6.17 × 6.38	N.S.
Perceived acidity/sweetness balance	6.82 × 6.44	N.S.	6.82 × 7.01	N.S.	6.44 × 7.01	0.047
Fruit weight (g)	19.26 × 28.85	0.041	19.26 × 42.86	0.0021	28.58 × 42.86	0.019
Fruit height (mm)	31.59 × 43.79	0.0013	31.59 × 45.94	0.0008	43.79 × 45.94	N.S.
Fruit width (mm)	29.37 × 33.07	N.S.	29.37 × 38.75	0.0022	33.07 × 38.75	0.0051
Fruit thickness (mm)	27.89 × 32.73	0.025	27.89 × 37.62	0.0006	32.73 × 37.62	0.0090
Pedicel length (mm)	16.06 × 18.70	N.S.	16.06 × 18.77	N.S.	18.70 × 18.77	N.S.
Soluble solids content (°Brix)	19.18 × 18.27	N.S.	19.18 × 17.92	N.S.	18.27 × 17.92	N.S.

Only *p*-values lower than or equal to 0.05 are shown (rounded to two significant digits); the others are presented as non-significant (N.S.).

## Data Availability

The original contributions presented in this study are included in the article/Supplementary Material. Further inquiries can be directed to the corresponding author.

## Supplementary Materials

The following supporting information can be downloaded at https://www.mdpi.com/article/10.3390/plants15132095/s1: Supplementary Table S1: Parentage analysis; Supplementary Table S2: DAPC+dendrograms; Supplementary Table S3: Bruvo’s distance; Supplementary Table S4: Coefficient of variation; Supplementary Table S5: Phenotypes.
